# Retinol Binding Protein 4 reactivates latent HIV-1 by triggering canonical NF-κB, JAK/STAT5 and JNK signalling

**DOI:** 10.1038/s41392-025-02424-3

**Published:** 2025-10-03

**Authors:** Chiara Pastorio, Khumoekae Richard, Shariq Usmani, Ann-Kathrin Kissmann, Grigory Bolotnikov, Guillermo Gosálbez, Manuel Hayn, Lennart Koepke, Alina Sauertnik, Andrea Preising, Nico Preising, Ludger Ständker, Matthew Fair, Jessicamarie Morris, Emmanouil Papasavvas, Qin Liu, Honghong Sun, Armando Rodríguez, Karam Mounzer, Sebastian Wiese, Pablo Tebas, Yangzhu Du, Gregory M. Laird, Markus Jaritz, Frank Rosenau, Moritz M. Gaidt, Konstantin M. J. Sparrer, Luis J. Montaner, Frank Kirchhoff

**Affiliations:** 1 Institute of Molecular Virology, Ulm University Medical Centre, Ulm, Germany; 2 HIV Cure and Viral Diseases Center, The Wistar Institute, Philadelphia, PA USA; 3 Institute of Pharmaceutical Biotechnology, Ulm University, Ulm, Germany; 4 Core Facility Functional Peptidomics (CFP), Ulm University Medical Center, Ulm, Germany; 5 Department of Pathology and Laboratory Medicine, Perelman School of Medicine, University of Pennsylvania, Pennsylvania, PA USA; 6 Core Unit Mass Spectrometry and Proteomics (CUMP), Ulm University Medical Center, Ulm, Germany; 7 AccelevirDx, Baltimore, MD USA; 8 Research Institute of Molecular Pathology, Vienna BioCenter, Vienna, Austria; 9 German Center for Neurodegenerative Diseases (DZNE), Ulm, Germany

**Keywords:** Preclinical research, Infectious diseases

## Abstract

Reactivation of the latent viral reservoirs is crucial for a cure of HIV/AIDS. However, current latency reversing agents are inefficient, and the endogenous factors that have the potential to reactivate HIV in vivo remain poorly understood. To identify natural activators of latent HIV-1, we screened a comprehensive peptide/protein library derived from human hemofiltrate, representing the entire blood peptidome, using J-Lat cell lines harboring transcriptionally silent HIV-1 GFP reporter viruses. Fractions potently reactivating HIV-1 from latency contained human Retinol Binding Protein 4 (RBP4), the carrier of retinol (Vitamin A). We found that retinol-bound holo-RBP4 but not retinol-free apo-RBP4 strongly reactivates HIV-1 in a variety of latently infected T cell lines. Functional analyses indicate that this reactivation involves activation of the canonical NF-κB pathway and is strengthened by JAK/STAT5 and JNK signalling but does not require retinoic acid production. High levels of RBP4 were detected in plasma from both healthy individuals and people living with HIV-1. Physiological concentrations of RBP4 induced significant viral reactivation in latently infected cells from individuals on long-term antiretroviral therapy with undetectable viral loads. As a potent natural HIV-1 latency-reversing agent, RBP4 offers a novel approach to activating the latent reservoirs and bringing us closer to a cure.

## Introduction

HIV-1 is the main causative agent of AIDS, a pandemic that has already affected more than 80 million people and led to an estimated 42.3 million deaths as of 2024 (Global HIV Statistics 2024, UNAIDS). HIV-1 infections are characterized by progressive depletion of CD4+ T-helper cells, chronic immune activation, and increased susceptibility to opportunistic infections.^1^ Combined antiretroviral therapy (cART) prevents HIV-1 replication and has greatly improved the life expectancy and quality of infected individuals.^2^ However, cART fails to cure HIV infection, and treatment is required for life. A reason for this is the livelong persistence of the virus in a small percentage of resting CD4+ T cells even under effective cART.^3,4^ These cells carry stably integrated and transcriptionally silent but still replication-competent proviruses in a reversibly non-productive state of infection. While they do not produce virus particles in this resting state, they can be reactivated to produce virions that may cause spreading infection and disease upon treatment interruption.^5,6^ This latent reservoir is already established early during acute HIV-1 infection and remarkably stable, even under long-term effective therapy. In addition to residual viral replication, it is also maintained through homeostatic proliferation and clonal expansion of infected cells. Targeting and eradicating the latent viral reservoirs is essential for achieving a cure for HIV/AIDS.

One therapeutic strategy to reduce HIV-1 reservoir size is the so-called “kick (or shock) and kill” approach.^7,8^ The first step involves the administration of latency-reversing agents (LRAs) to people living with HIV (PLWH) on cART. These agents aim to “kick” the virus out of latency by reactivating transcriptionally silent HIV-1 proviruses in infected cells. Once HIV-1 proviruses are reactivated, the infected cells begin producing viral proteins and particles, rendering them visible to the immune system and susceptible to clearance through cytolytic immune responses, such as cytotoxic T lymphocytes (CTLs) and natural killer (NK) cells. At the same time, de novo infection of cells is prevented by cART.^9^ Several classes of compounds have been characterized for their ability to induce reactivation of HIV-1 gene expression from latency, including protein kinase C (PKC) agonists, histone deacetylase inhibitors (HDACi), histone methylation inhibitors (HMTi), and DNA methyltransferase inhibitors.^9^ Despite promising in vitro results, current “kick and kill” strategies have thus far failed to significantly reduce the latent HIV-1 reservoirs in clinical trials.^10^ Limitations include insufficient potency, poor selectivity, and the inability to elicit robust immune-mediated clearance of reactivated cells, highlighting the need for improved LRAs and combination therapies to enhance both the reactivation and killing parts.^10^

Upon interruption of cART, HIV-1 rebounds to detectable levels in most people living with HIV (PLWH) within a few weeks.^11^ Endogenous cytokines, such as TNF-α (tumor necrosis factor-alpha) and IL-1β (interleukin-1 beta), can act as latency-reversing agents by activating NF-κB signaling, which in turn promotes HIV-1 transcription. Other innate immune stimuli and proinflammatory cytokines may also contribute to reactivation, especially under conditions of immune activation, such as co-infections, tissue injury, or auto-inflammatory diseases. Altogether, however, it is currently poorly understood which endogenous factors reactivate HIV-1 from latency. Their identification may not only help to better understand the mechanisms underlying viral rebound but also provide novel means to drive HIV-1 out of latency and reduce the size of the latent viral reservoirs. Moreover, understanding the context and tissue microenvironments in which endogenous reactivation occurs may help to develop interventions that prevent rebound or increase clearance of reactivated HIV-producing cells.

Here, we performed a systematic screen of a hemofiltrate-derived peptide library for endogenous circulating activators of latent HIVs in J-Lat cells. Hemofiltrate can be obtained in large quantities from patients with chronic renal failure and is a valuable source of biologically active peptides.^12^ Peptide libraries derived from hemofiltrate are estimated to contain over one million distinct peptides and small proteins, encompassing virtually all circulating molecules with a molecular weight below 30 kDa. These include chemokines, defensins, cytokines, and hormones, present in a highly concentrated and bioactive form.^12^ Importantly, the relative concentrations of these compounds largely reflect those found in human plasma, making hemofiltrate-derived libraries physiologically relevant. Previous studies have shown that these peptide libraries are a valuable resource for the identification and isolation of novel natural compounds with anti-HIV-1 activity.^13^ The J-Lat latency model is based on various subclones of Jurkat T cells containing transcriptionally silent HIV-1 proviruses carrying the GFP reporter gene^14^ and has been extensively used to study viral latency and reactivation.^15–17^ We discovered that human Retinol Binding Protein 4 (RBP4) carrying retinol (vitamin A) strongly activates latent HIV-1 in J-Lat 10.6, 11.1, A1 and H2 cell lines. In agreement with a relevant role in vivo, holo-RBP4 also reactivated HIV-1 in cells obtained from people living with HIV (PLWH) after long-term antiretroviral therapy. Our data support that the abundant carrier of retinol RBP4 promotes HIV-1 expression and offers prospects for reactivation of the latent viral reservoirs.

## Results

### Identification of RBP4 as activator of latent HIV-1 proviruses

**Fig. 1:**
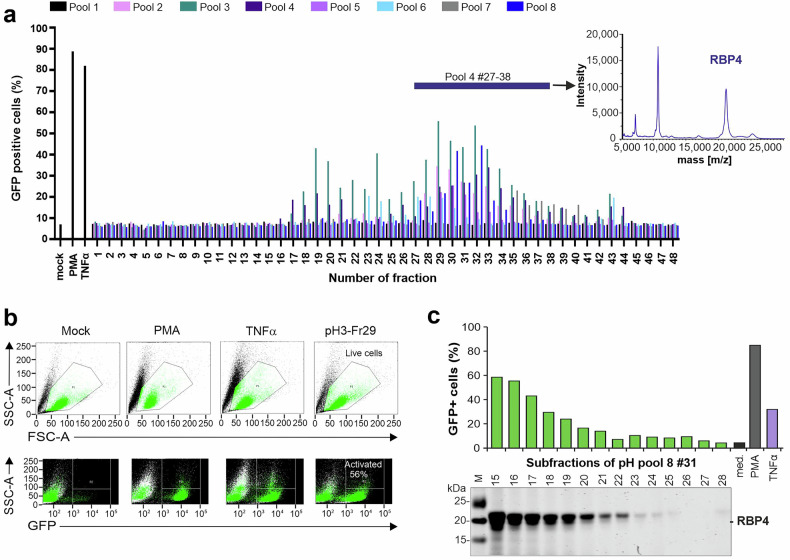
Identification of RBP4 as an enhancer of latent HIV. **a** (Left panel) Activation of latent HIV-1 eGFP reporter proviruses in J-Lat 11.1 cells treated with peptide fractions of a hemofiltrate (HF)-derived library. The color of the bar indicates to which pool of the peptide library the fraction belongs. Mock indicates the absence of peptide fractions; PMA and TNFα were used as positive controls. Fractions highlighted by the upper bar were used for further purification. (Right panel) MALDI-TOF spectrum of RBP4 shows a dominant signal of *m*/*z* 20,960.75, which closely matches the theoretical *m*/*z* value of the RPB4 (1-182, 20,959.42 Da) truncated variant lacking the C-terminal leucin. **b** Example for activation of latent HIV-1 in J-Lat 11.1 cells by a single HF-derived fraction compared to the PMA and TNFα controls, determined by flow cytometry. **c** The levels of RBP4 in subfractions of the pool 8 HF fraction 31 detected by SDS-PAGE after purification (lower panel) correlate with the percentages of cells showing reactivation of HIV-1 eGFP reporter proviruses

To identify endogenous circulating factors that reactivate latent HIV-1, we generated a protein/peptide library from human hemofiltrate (HF) using cation exchange (generating 8 pH pools) and reversed phase (RP) chromatography, resulting in 48 fractions per pool.^13^ As previously reported,^13^ these libraries contain essentially all peptides and small proteins circulating in human blood in a lyophilized, bioactive and highly concentrated form. The total of 384 peptide-containing RP-HPLC fractions from the HF peptide library were analyzed for factors reactivating latent proviruses (i.e. are inducing GFP expression) in J-Lat 11.1 cells, which carry an *env*-defective HIV-1 construct containing the *GFP* reporter gene in place of the accessory *nef* gene.^14^ Phorbol myristate acetate (PMA) and tumor necrosis factor-alpha (TNF-α), two well-established activators of HIV-1 latency, were used as positive controls. A strikingly high number of HF fractions from different pH pools reactivated HIV-1 transcription (Fig. 1a). In several cases, the levels of GFP expression were about as high as those achieved with PMA and TNF-α (example shown in Fig. 1b), which are highly potent but impractical for therapeutic use due to high toxicity and nonspecific activation of immune cells. Fractions 27 to 38 of pH pool 4 that reactivated latent HIV were combined and further separated by additional chromatographic steps (Supplementary Fig. 1a). SDS-PAGE revealed that active fractions contained a protein of ~21 kDa (Supplementary Fig. 1b). MALDI-TOF MS analysis of the activating fraction F42 obtained after the final purification step revealed the mono, double and triple charged species of Retinol Binding Protein 4 (RBP4) (Fig. 1a and Supplementary Fig. 1c, d), a member of the lipocalin family.^18^ Specifically, the species identified (*m*/*z* 20,960.76) corresponds to RBP4 lacking the C-terminal leucine (RBP4 1-182). Notably, forms of RBP4 lacking the C-terminal leucine have been previously detected in serum of hemolysis patients.^19^ The N-terminal secretory signal peptide of 18 amino acids (aa) is cleaved upon processing, and RBP4 circulates as a protein of 183 amino acids in human blood.^20^ RBP4 is the main carrier of Vitamin A (Retinol) and transports it to different target tissues.^21^ In addition, RBP4 contributes to insulin resistance in obesity and type 2 diabetes and may induce TLR4 signaling in some immune cells.^21,22^ RBP4 was readily detectable in active HF fractions (Fig. 1c) and its concentrations correlated with the levels of HIV-1 reactivation indicated by the percentages of GFP+ cells (*R*^2^ = 0.667; *p* < 0.0001) (Supplementary Fig. 1e). Thus, our results identified RBP4 as potent circulating activator of latent HIV-1 proviruses.

### RBP4 activates latent HIV-1 in a variety of T cell lines

**Fig. 2:**
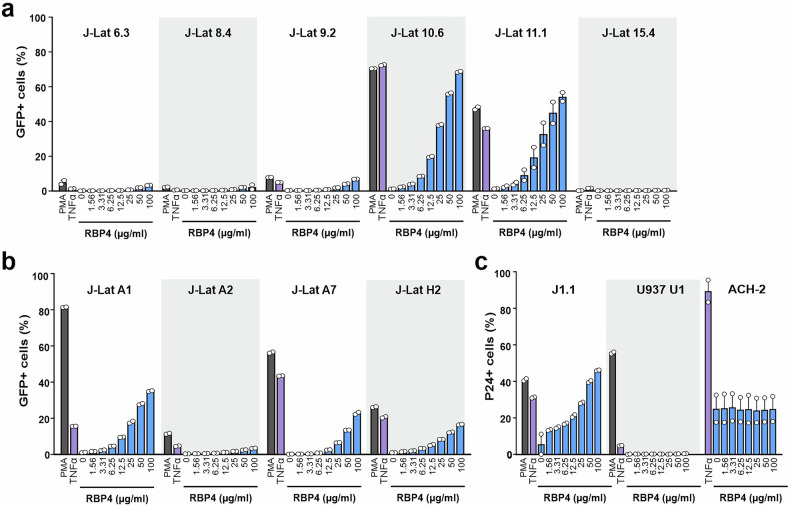
RBP4-mediated reactivation of latent HIV-1 in various cell lines. J-Lat cell lines harboring **a** full-length HIV-1 eGFP proviruses or **b** LTR-Tat-IRES-GFP cassettes were treated with the indicated concentrations of RBP4 or PMA and TNFα for control and the percentages of green cells were determined by flow cytometry. **c** Jurkat J1.1 cells, U937 and ACH-2 cells harboring full-length intact HIV-1 proviruses were treated as in **a** and **b** but reactivation was determined by intracellular p24 antigen staining. Data are represented as mean ± SEM of two independent experiments

Our initial screen was performed using the J-Lat 11.1 subclone that is highly responsive to agents inducing latent HIV-1 reactivation.^14,23^ A variety of J-Lat cell lines harboring either proviral sequences containing GFP in place of *nef* or LTR-Tat-IRES-GFP expression cassettes thought to be silenced by different mechanisms are available.^14^ To assess the spectrum of latency reversal activity, we examined the effect of RBP4 from hemofiltrate on 10 different J-Lat subclones. RBP4 dose-dependently and efficiently reactivated latent HIV-1 proviruses in the 10.6 and 11.1 cell lines but had no or only modest effects in the J-Lat 6.3, 8.4, 9.2 and 15.4 lines (Fig. 2a). Similarly, RBP4 enhanced GFP expression in the A1, A2 and H2 J-Lat Tat GFP reporter cell lines, while little induction of GFP expression was observed in A7 J-Lat cells (Fig. 2b). In addition, RBP4 also induced p24 production in the Jurkat J1.1 T cell clone that harbors the HIV-1 LAV-1 strain and has defects in CD3 signaling (Fig. 2c).^24^ In contrast, RBP4 did not significantly stimulate p24 antigen expression in the chronically HIV-1 LAV-1-infected promonocytic U937 cell line derivative U1 and in the HIV-1 LAV-1 latently infected lymphocytic CEM cell line ACH-2 (Fig. 2c).^25^ Overall, RBP4 induced HIV-1 reactivation from latency to similar levels as PMA or TNFα in about half of the model cell lines tested.

### Retinol is required but not sufficient for HIV-1 reactivation

**Fig. 3:**
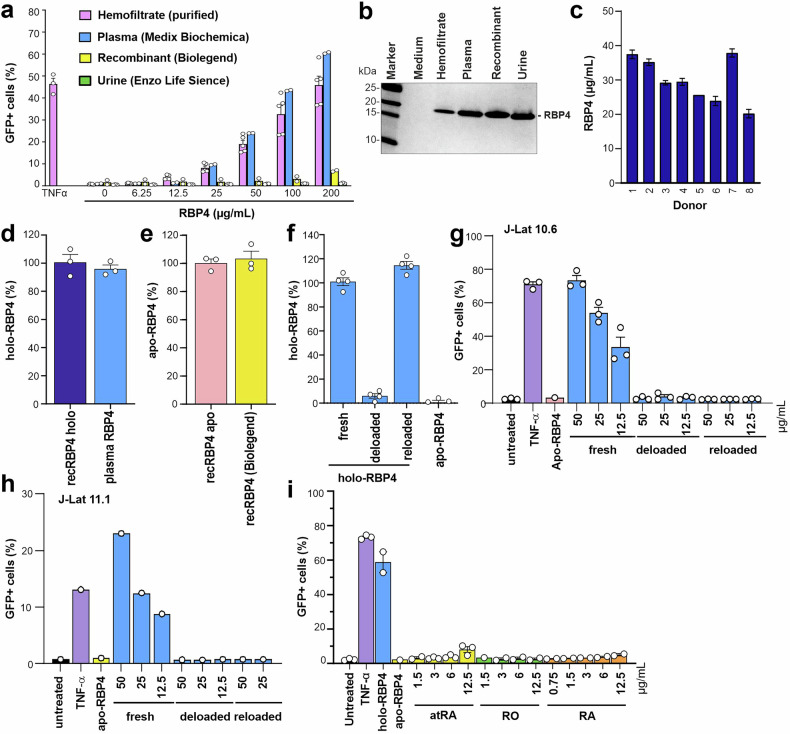
Impact of RBP4 from different sources and free retinol and its metabolic products on HIV-1 reactivation. **a** RBP4 from the indicated sources was tested for its ability to reactivate latent HIV-1 in the J-Lat 10.6 cell line at the indicated concentrations and examined by SDS-PAGE (**b**). TNFα was used as a positive control. Data are represented as mean ± SEM of two to five independent experiments. **c** Concentration of RBP4 in the plasma from eight healthy donors measured by ELISA assay. Data are represented as mean ± SD of two technical replicates. **d** Percentage of holo-RBP4 in plasma holo-RBP4 (Medix Biochemica), based on intrinsic fluorescence (Ex: 280 nm/Em: 340 nm). Recombinant holo-RBP4 is shown as a positive control for comparison. Data are represented as mean ± SEM of three independent experiments. **e** Percentage of apo-RBP4 in recombinant human apo RBP4 (Biolegend), based on intrinsic fluorescence (Ex: 280 nm/Em: 340 nm). Recombinant apo-RBP4 is shown as a positive control for comparison. Data are represented as mean ± SEM of three independent experiments. **f** Kinetics of retinol loading into deloaded apo-RBP4 measured by a fluorescence decrease, indicating reconstitution of the holo state. Data are represented as mean ± SEM of four independent experiments. **g** J-Lat 10.6 cells were treated with the indicated concentrations of fresh, deloaded or reloaded holo-RBP4 (Medix Biochemica) and the percentage of GFP-positive cells was determined by flow cytometry. TNF-α and apo-RBP4 (Biolegend) are shown for comparison. Data are represented as mean ± SEM of three independent experiments. **h** J-Lat 11.1 cells were treated with the indicated concentrations of fresh, deloaded or reloaded holo-RBP4 (Medix Biochemica) and the percentage of GFP-positive cells was determined by flow cytometry. TNF-α and apo-RBP4 (Biolegend) are shown for comparison. **i** J-Lat 10.6 cells were treated with the indicated concentrations of all trans-retinal (atRA), retinol (RO) and retinoic acid (RA) and the percentage of GFP-positive cells was determined by flow cytometry. TNFα, holo-RBP4 and apo-RBP4 are shown for comparison. Data are represented as mean ± SEM of two to three independent experiments

To obtain further insights into its latency reversing effects, we analyzed RBP4 purified from four different sources: hemofiltrate, human blood plasma (Medix Biochemica), human urine (Enzo Life Sciences), or recombinant expression (Biolegend). We found that RBP4 purified from hemofiltrate in our laboratory or from blood plasma efficiently reactivated latent HIV-1 in J-Lat 10.6 cells (Fig. 3a). In contrast, recombinant RBP4, as well as RBP4 isolated from human urine, displayed little if any activity, although proteins of correct size were readily detectable on SDS-PAGE (Fig. 3b). Treatment of J-Lat cells with LPS did not induce HIV-1 or significant proinflammatory cytokine expression (Supplementary Fig. 2), excluding endotoxin contamination as a cause of latency reactivation. In agreement with published data,^26^ the levels of RBP4 in plasma from healthy individuals determined by ELISA varied between 20 and 40 µg/ml (Fig. 3c). These concentrations were sufficient to reactivate latent HIV-1 in a variety of model cell lines (Fig. 2). Thus, RBP4 is capable of reactivating HIV-1 from latency at physiological concentrations.

The main function of RBP4 is to transport retinol (vitamin A) from the liver to peripheral tissues. Two different isoforms of RBP4 exist in serum, namely holo-RBP4 (Retinol-bound RBP4) and apo-RBP4 (Retinol-free RBP4).^26^ RBP4 largely circulates in complex with transthyretin (TTR) to prevent its loss from the circulation by filtration in the renal glomeruli.^27^ After delivery of vitamin A, RBP4 loses the affinity for TTR and apo-RBP4 is filtered through the glomeruli of the kidneys and ends up in urine. Since retinol has a very low molecular weight (0.286 kDa), these two forms migrate at a similar size in SDS-PAGE. In agreement with previous data,^27,28^ a fluorescence-based assay^29^ confirmed that plasma-derived RBP4 contained retinol (Fig. 3d), while inactive recombinant RBP4 lacked it entirely (Fig. 3e). For control, recombinantly produced apo-RBP4 and freshly loaded holo-RBP4 were produced and characterized as previously described.^30^ To further assess the requirement of retinol for latency reactivation, we depleted retinol from active plasma-derived holo-RBP4 and subsequently reloaded it (Fig. 3f and Supplementary Figs. 3, 4). Efficient depletion and reloading were confirmed by both fluorescence and reduced graphene-oxide-based field effect transistors (rGO-FETs) functionalized with aptamers against one of the RBP4 forms (Supplementary Figs. 4 and 5).^30^ Removal of retinol entirely abolished the ability of RBP4 to reactivate latent HIV-1 in both J-Lat 10.6 and 11.1 cells (Fig. 3g, h). However, reloading did not restore this activity, possibly because in vitro assembled holo-RBP4 may differ from circulating holo-RBP4 that is co-assembled and released with TTR.^31,32^

It has been reported that retinoic acid (RA), the biologically active metabolite of retinol, reactivates latent HIV-1 proviruses.^33,34^ To determine whether retinol or its metabolites are sufficient for reactivation, we examined the effects of purified free forms of retinoids. All trans-retinal (atRA), retinol and RA all had little effect on HIV-1 latency reactivation in J-Lat 10.6 cells, while holo-RBP4 was highly effective (Fig. 3i). In addition, atRA was cytotoxic at higher concentrations (Supplementary Fig. 6a). Uptake of free unbound RA is typically minimal under physiological conditions and most RA in cells is generated from the delivery of retinol by RBP4 upon binding of the receptor ’stimulated by retinoic acid 6’ (STRA6) and subsequent conversion to RA within the cells. To exclude that poor uptake was the reason for lack of proviral reactivation by free retinol and RA, we treated J-Lat 10.6 cells with DEAB or WIN18,446, which inhibit the conversion of retinal to retinoic acid upon delivery by holo-RBP4.^35^ Both agents had no inhibitory effect on reactivation of HIV-1 by TNF-α or RBP4 (Supplementary Fig. 6b). Altogether, these results are strong evidence that HIV-1 reactivation requires the circulating holo form of RBP4 but is independent of retinoic acid production in the target cells.

### Holo-RBP4 induces HIV-1 and NF-κB signaling genes in J-Lat 10.6 but not in 8.4 cells

**Fig. 4:**
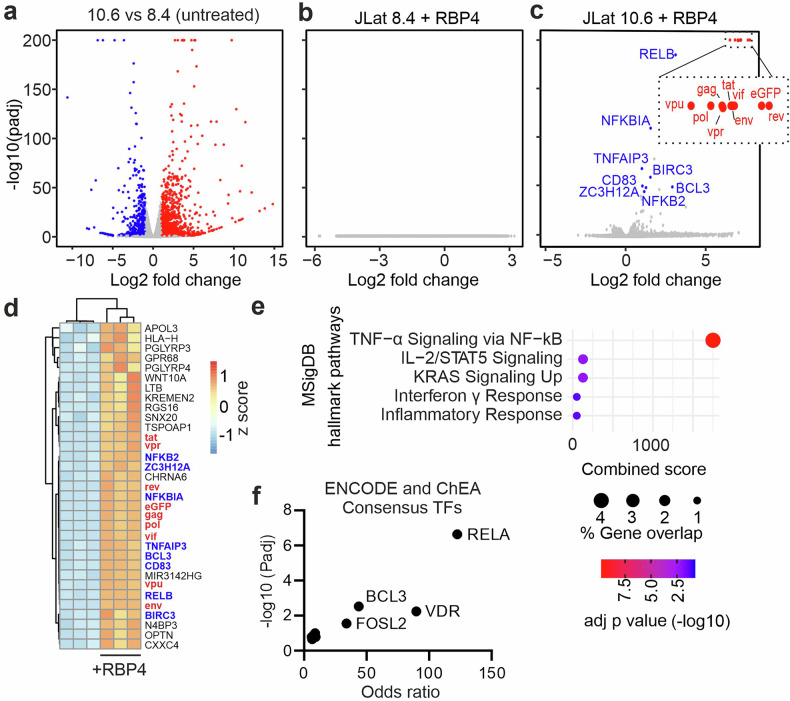
Transcriptomic response of J-Lat 8.4 and 10.6 cells to holo-RBP4. **a** Volcano plot depicting the differentially expressed genes in untreated J-Lat 10.6 versus 8.4 cells as assessed by RNAseq and deseq2. Log2 fold change of *N* = 3 (biological replicates), to control he false discovery rate significances given as threshold-adjusted *p* values. Volcano plot of the differential expressed genes in **b** J-Lat 8.4 or **c** 10.6 cells treated with holo-RBP4 as assed by RNAseq and deseq2 analysis. Log2 fold change of *N* = 3 (biological replicates), to control the false discovery rate significances given as threshold-adjusted *p* values. **d** Heatmap depicting the top 40 differentially regulated genes in J-Lat 10.6 cells, individual biological replicates treated as indicated. Genes and replicates are grouped by hierarchical clustering and the expression values *z*-score normalized. **e** Gene Ontology Term enrichment analysis driven by EnrichR of the significantly upregulated genes in (**c**). **f** Chip-X prediction of the major transcription factors driving expression of the significant genes in (**c**)

To obtain insights into the mechanism(s) of RBP4-mediated HIV-1 reactivation, we analyzed the transcriptional response to RBP4 by RNA-seq in J-Lat 8.4 and 10.6 cells (Fig. 4a). Holo-RBP4 treatment did not induce any significant transcriptional changes in J-Lat 8.4 cells (Fig. 4b). In contrast, effective viral gene expression and a limited number of cellular genes were induced in J-Lat 10.6 cells (Fig. 4c, d). EnrichR gene ontology analysis revealed that these factors mainly belong to “TNF-α signaling via NF-κB” (Fig. 4e). Among them were several key components of the non-canonical NF-κB pathway: RELB forms active transcriptional complexes with p52 (from NFKB2), while BIRC3 regulates NIK stability to control pathway activation. Additional factors included NFKBIA, TNFAIP3, and BCL3, which modulate p50/p52 activity. Two NF-κB target genes (CD83 and ZC3H12A) involved in immune regulation and feedback inhibition were also induced. To identify transcription factors involved in RBP4-mediated gene induction, we performed Enrichr-based analysis of RBP4-upregulated genes in J-Lat 10.6 using ENCODE and ChEA consensus TF annotations (Fig. 4f). The results suggest that the canonical NF-κB factor RELA likely drives the induction of this specific gene set. Notably, RBP4 did not induce TNF-α secretion in J-Lat 10.6 cells, and unlike TNF-α, failed to reactivate HIV-1 in ACH-2 cells (Fig. 2). Altogether, RBP4 and TNF-α exhibited overlapping but distinct abilities to reactivate latent HIV.

### RBP4-mediated HIV-1 reactivation does not correlate with STRA6 mRNA expression

To explore the basis for the differential responsiveness of J-Lat cell lines to RBP4, we compared their mRNA expression profiles in the absence of treatment. Altogether, J-Lat 8.4 and 10.6 cells differed in the expression of ~2000 genes (Fig. 4a). STRA6, the primary receptor for holo-RBP4,^36^ and TLR4, which mediates RBP4-induced immune signaling in macrophages,^37^ were evaluated as potential determinants. However, J-Lat cells lacked TLR4 expression (Supplementary Fig. 7a). Moreover, both RBP4-responsive (10.6 and 11.1) and non-responsive (8.4) J-Lat clones expressed similar levels of STRA6 mRNA (Supplementary Fig. 7b). STRA6 expression was very low or undetectable in activated and non-activated CD4+ T cells from healthy donors (Supplementary Fig. 7b). RBP4 treatment did not induce IL-6, IL-1β, or STRA6 expression in J-Lat lines, regardless of HIV-1 reactivation responsiveness (Supplementary Fig. 7b, c). Finally, knockout of STRA6 (by ~56%; Supplementary Table 1) did not affect RBP4-mediated HIV-1 reactivation in 10.6 cells (Supplementary Fig. 8). These findings suggest that holo-RBP4 reactivates latent HIV-1 via an as-yet-unknown receptor.

### RBP4-induced HIV-1 reactivation involves NF-κB, JAK/STAT5 and JNK signaling

**Fig. 5:**
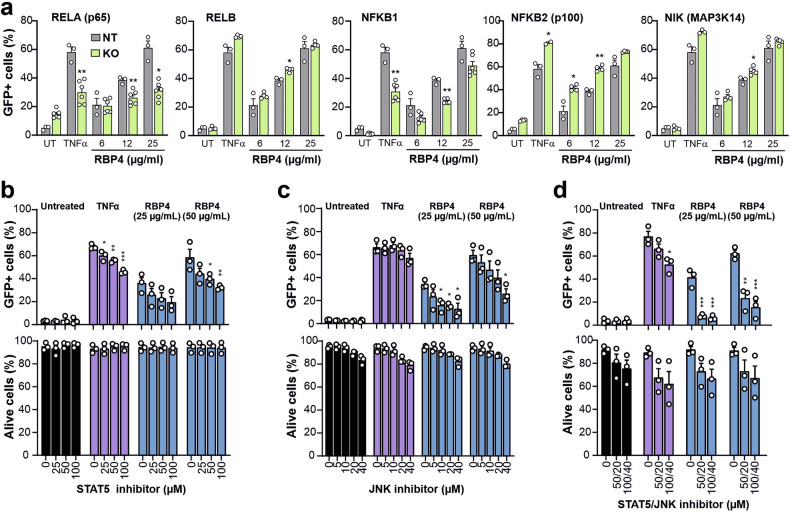
Role of JAK/STAT and TLR/JNK activation in RBP4-mediated HIV reactivation. **a** Reactivation of HIV-1 eGFP reporter proviruses in NT control (gray) or RELA, RELB, NFKB1, NFKB2 or NIK (green) KO J-Lat 10.6 cells treated with the indicated concentrations of holo-RBP4, TNFα or left untreated. Data are represented as mean ± SEM of three independent experiments with the NT sgRNA (gray) or two different targeting sgRNAs (green). *p* values are shown as measured by two-tailed Student’s *t* test with Welch’s correction. J-Lat 10.6 cells were pretreated with the indicated concentrations of STAT5 (**b**) or JNK (**c**) inhibitor or their combination (**d**). Four hours later, the cells were left untreated or treated with TNFα or the indicated concentrations of holo-RBP4. The percentage of cells showing reactivation of HIV-1 eGFP reporter proviruses (upper panel) and the cell viability (lower panel) were determined by flow cytometry. Data are represented as mean ± SEM of three independent experiments. *p* value are shown as measured by one-way ANOVA

To further define the cellular factors involved in latency reactivation by RBP4, we performed comprehensive knock-out (KO) and inhibition studies. KO of factors involved in canonical NF-kB signaling (RELA by ~65–81%, NFKB1 by ~32–55%; Supplementary Table 1 and Supplementary Fig. 9) clearly reduced HIV-1 reactivation (Fig. 5a). In contrast, KO of factors involved in the non-canonical NF-κB pathway (RELB, NFKB2, NIK) had no or even moderately enhancing effects. In line with this, inhibition of canonical NF-κB signaling by BAY 11-7082 reduced RBP4-mediated HIV reactivation but also showed significant cytotoxicity, while two inhibitors of the non-canonical pathways had no inhibitory effect (Supplementary Fig. 10). Previous studies support that HIV-1 reactivation in J-Lat systems depends on the activation of canonical NF-κB activation as an initial trigger, followed by modulation of JAK/STAT5 and JNK (MAPK) pathways for robust and sustained HIV-1 reactivation.^16,38,39^ In agreement with a cooperative role of these pathways, treatment with a STAT5 inhibitor (Biomol) reduced RBP4-mediated reactivation of HIV-1 from latency in a dose-dependent manner without causing toxic effects (Fig. 5b and Supplementary Fig. 11a). Similarly, treatment with a JNK inhibitor dose-dependently decreased RBP4-mediated reactivation of latent HIV-1 by up to 70% (Fig. 5c and Supplementary Fig. 11b). Since holo-RBP4 has been reported to exert complex effects on cell metabolism and signaling,^40^ we also examined inhibitors of PKC (Rottlerin), ERK1/2 (U0126) and MEK1/2 (Selumetinib) signaling. However, none of these agents significantly affected HIV-1 reactivation (Supplementary Fig. 12). In contrast, combined treatment with a STAT5 and JNK inhibitor clearly reduced the induction of proviral gene expression by RBP4 (Fig. 5d). To assess possible synergistic effect of JAK/STAT5 and JNK signaling in RBP4-induced HIV-1 reactivation, we examined the effects of inhibitors in J-Lat 10.6 cells after KO of a variety of factors (with an efficiency of ~52–90%). KO of STAT5A and MAPK9 (JNK2) moderately reduced RBP4-mediated latent HIV activation in both the presence and absence of JAK/STAT5 and JNK inhibitors, while KO of STAT5B and MAPK8 (JNK1) had no significant effect (Supplementary Fig. 13). The inhibitors still had an additional impact in KO cells, most likely due to incomplete KOs (Supplementary Table 1), as clonal cell lines could not be generated due to time constraints. Furthermore, the inhibitors have broader activity—for example, the JNK inhibitor targets JNK1 in JNK2 KO cells. Altogether, our results support a model in which RBP4 activates latent HIV via initial triggering of the canonical NF-κB pathway, with additional contributions from JAK/STAT5 and JNK signaling, as KO of STAT5A and MAPK9 reduced reactivation efficiency.

### RBP4 reactivates latently infected cells derived from PLWH under ART

**Fig. 6:**
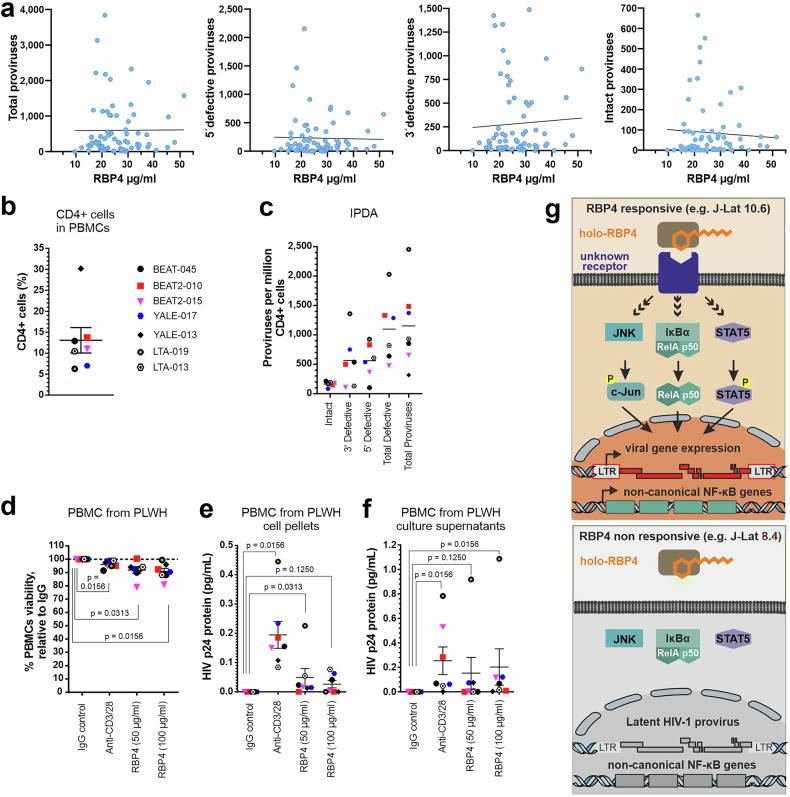
Reactivation of latent HIV-1 in PBMCs from PLWH under cART. **a** Correlation between the number of total, defective and intact proviruses and RBP4 plasma levels in the corresponding PLWH using the Spearman correlation test. **b** Percentage of CD4+ cells of PBMCs from 7 PLWH under cART. **c** Percentage of intact and defective proviruses in CD4+ cells from cART-suppressed study participants, measured through IPDA. **d** Percent PBMC viability relative to IgG control after 72 h treatment with LRAs. Detection of Gag-p24 HIV-1 protein in cART-suppressed PBMC cell pellets (**e**) and culture supernatants (**f**) after 72 h treatment with LRAs as measured by SIMOA. *p* values are shown as measured by the Wilcoxon Signed-Rank test. **g** Proposed model of holo-RBP4-mediated reactivation of latent HIV-1. RBP4 activates latent HIV in responsive cells through an unknown receptor, independent of TLR4 and STRA6, which are absent or similarly expressed in both responsive and non-responsive cells. Inhibition and gene knockout studies implicate the canonical NF-κB, JNK, and STAT pathways in HIV reactivation. The coordinated engagement of these pathways leads to efficient reactivation of latent HIV. In addition, holo-RBP4 selectively induces a limited set of host genes, including the non-canonical NF-κB pathway in responsive cells, suggesting a distinct transcriptional signature associated with reactivation. The image has been generated using CorelDraw 2021.5

The levels of RBP4 are increased in various inflammatory diseases^21,41^ and inflammatory mediators are frequently enhanced in PLWH even after long-term ART.^42^ To determine the levels of RBP4 and assess potential correlations with the size of the latent viral reservoirs, we analyzed these parameters in a total of 64 ART-treated PLWH. The RBP4 levels ranged from about 10 to 50 µg per ml in plasma (Fig. 6a), which agrees with the circulating concentrations found (Fig. 2c) and reported for healthy adults.^26^ They did not correlate with the numbers of total, defective or intact proviruses in the corresponding individuals (Fig. 6a). However, the average concentrations (26.25 ± 8.68 µg/ml) were well sufficient for significant reactivation of HIV-1 in in vitro latency models.

To assess the potential relevance of RBP4 expression in vivo, we examined its effect on PBMCs from seven people living with HIV (PLWH). All study participants were male and all but one African Americans (Table 1). The majority of them were under effective ART for more than 10 years and had undetectable viral loads at the day of blood collection. CD4+ T cells were in the normal range (Table 1 and Fig. 6b). Using the well-established Intact Proviral DNA Assay,^43^ we also assessed the composition and levels of intact and defective proviruses in CD4+ T cells from each donor. Proviral sequences were detectable in all individuals, but their levels varied substantially (Fig. 6c), with the frequency of intact HIV-1 proviruses being generally lower than the defective proviruses, consistent with previous data.^44^ To measure ex vivo latency reversal activities of RBP4, we next treated the PBMCs from the seven virally suppressed study participants with RBP4 (50 and 100 µg/mL), alongside anti-CD3/CD28 beads (50 µg/mL) as a positive control and Immunoglobulin G (IgG) (50 µg/mL) as the negative control for 72 h, and measured the expression of HIV Gag-p24 protein in the cell pellets and culture supernatants using the ultra-sensitive Single Molecule Array (SIMOA).^45^ RBP4 was generally tolerated by the cells as no extensive toxicity was detected relative to the IgG vehicle control (Fig. 6d). Our results show that RBP4 significantly reactivates latent HIV-1 in PBMCs from cART-suppressed individuals (*p* < 0.05), even though its potency was inferior to the anti-CD3/28 positive control (Fig. 6e, f). IPDA analyses indicate that induction of p24 expression by RBP4 correlated with the number of total and defective but not intact proviruses (Supplementary Fig. 14). Altogether, the results show that RBP4 has a significant impact on HIV latency reactivation in cells from PLWH under effective ART at physiological concentrations.

**Table 1:** Study participants baseline characteristics

Patient ID	Gender	Age	Ethnicity	Race	Years since HIV diagnosis	Years on ART	Years of continual viral suppression	CD4 nadir recorded at first diagnosis	Current ART regimen	CD4 Count on day of collection	Viral load Count on day of collection
BEAT-045	Male	45	Not Hispanic	AA	24	18	11	77	Triumeq	573	<20
BEAT2-010	Male	60	Not Hispanic	White	24	23	2	>200	Biktarvy	1219	<20
BEAT2-015	Male	62	Not Hispanic	AA	27	20	12	>200	Triumeq	396	<20
Yale-017	Male	43	Not Hispanic	AA	19	19	12	349	Symtuza	653	51
Yale-013	Male	57	Not Hispanic	AA	20	18	17	425	Biktarvy	694	<20
LTA-019	Male	72	Not Hispanic	AA	35	20	18	126	Biktarvy	921	<20
LTA-013	Male	56	Not Hispanic	AA	34	13	13	372	Biktarvy	411	<20

## Discussion

The persistence of HIV-1 in transcriptionally silent latent form in long-living memory CD4+ T cells is one of the main barriers to a cure of HIV/AIDS. Here, we show that holo-RBP4, the carrier of Vitamin A in the bloodstream, reactivates latent HIV-1 in a variety of T cells lines as well as in a significant number of cells from people with HIV. Holo-RBP4 is abundant in the circulation and has been reported to be further increased in PLWH under effective ART.^46^ We found that a surprisingly high number of hemofiltrate-derived fractions reactivates latent HIV-1 and this activity correlated with the presence of intact and C-terminally truncated RBP4 proteins. Notably, the relative concentrations of RBP4 in the peptide/protein library largely correspond to those circulating in human blood.^12^ In agreement with a relevant role of RBP4 as modulator of HIV-1 latency in vivo, significant reactivation from latency was observed at physiological concentrations of 10 to 50 µg/ml and confirmed using PBMCs obtained from PLWH after long-term cART and with undetectable viral RNA loads.

Previous studies identified STRA6 and TLR4 as RBP4 receptors.^36,37^ However, our data show that J-Lat cells lack TLR4 expression, and STRA6 is expressed in both responsive and non-responsive J-Lat subclones, suggesting that RBP4-mediated HIV-1 reactivation may depend on an unknown receptor (Fig. 6g). HIV-1 reactivation involved the induction of a distinct set of genes that are part of the “TNF-α signaling via NF-κB” pathway and regulated by canonical NF-κB transcription factors, such as RelA (Fig. 4c–f). Altogether, our data suggest that NF-κB signaling may be the initial trigger of holoRBP4-induced HIV-1 reactivation in J-Lat cells but that JAK/STAT and JNK signaling contribute to efficient and sustained reactivation (Fig. 6g). Previous studies reported that proviral integration sites and chromatin landscape rather than differences in signaling cascades determine the efficiency of HIV-1 reactivation in different J-Lat clones in response to TNF-α, PKC agonists, HDAC- or BET-inhibitors.^17,47,48^ However, here we show that besides proviral integration sites and chromatin context, the induction of a transcriptional response clearly plays a role in RBP4-mediated HIV reactivation from latency in the J-Lat model.

Accumulating evidence suggests that retinoic acid (RA), the biologically active metabolite of retinol, reactivates latent lentiviruses in vitro and in vivo.^33,34,49^ In addition, treatment with the RA derivative acitretin was shown to enhance RIG-I signaling and HIV-1 transcription.^50^ Thus, it came as a surprise that we observed only minimal effects of RA treatment (Fig. 3i). This was not just due to inefficient uptake, since treatment of J-Lat cells with agents preventing retinal-to-RA conversion did not impair HIV-1 reactivation by holo-RBP4 (Supplementary Fig. 6). We found that hemofiltrate-purified or commercial plasma-derived holo-RBP4 reactivates latent HIV-1 in the J-Lat model, whereas recombinant or urine-derived apo-RBP4 was inactive. Removal of retinol from circulating holo-RBP4 completely abolished its ability to reactivate HIV (Fig. 3f–h). However, reloading did not restore this function. Altogether, these results indicate that delivery of retinol and subsequent oxidation to RA are not required for RBP4-mediated reactivation of HIV-1 in the J-Lat system. A key difference between reloaded and physiological blood-derived holo-RBP4 is that the latter is produced and circulates in complex with transthyretin, which plays a critical role in retinol transport and bioavailability.^21,32^ It is known that reloading allows efficient delivery of retinol,^51^ but no studies have directly compared circulating (plasma- or blood-derived) holo-RBP4 with holo-RBP4 regenerated in vitro from apo-RBP4 with respect to conformation and receptor interactions. It is thus tempting to speculate that the presence of TTR might induce changes in holo-RBP4 that are critical for its ability to reactivate HIV-1 from latency.

One important question is whether circulating RBP4 affects the latent HIV reservoirs in vivo. While RBP4 is mainly produced in the liver, it is also an adipocytokine linked to obesity and insulin resistance.^52^ Obese children and adult individuals with obesity and type 2 diabetes have significantly higher RBP4 levels compared with lean healthy controls.^53^ Notably, the mechanisms involved in reactivation of latent HIV-1, activation of NF-κB, JNK, and JAK/STAT signaling, contribute to the detrimental effects of RBP4 on insulin sensitivity.^29,37^ Thus, the latent HIV-1 reservoirs may be altered in obese PLWH. Although the plasma levels of RBP4 in PLWH were well sufficient to reactivate HIV-1 in latency models, they did not correlate with proviral loads in blood (Fig. 6a). This likely reflects the heterogeneity of the latent HIV reservoir, as only a subset of latently infected cells may be responsive to RBP4, while others remain resistant due to lack of the receptor or epigenetic constraints. In addition, other circulating factors, such as TNF-α and IL-1β, also affect the viral reservoirs in vivo.^54^ Altogether, the effects of holo-RBP4 on HIV replication, latency, and pathogenicity seem complex and warrant further investigation.

While our study clearly demonstrates that circulating holo-RBP4 reactivates HIV-1 in human T cell lines, as well as in cells derived from PLWH, it has some limitations. Cell culture models are useful for screening and mechanistic studies, but do not fully recapitulate the in vivo conditions. For example, the relative contributions of different RBP4 receptors and metabolites to HIV-1 reactivation in PLWH remain to be determined. The effect of holo-RBP4 on latent HIV reservoirs in individuals with obesity also requires further study. A key question is which unknown receptor may mediate the differential responsiveness to holo-RBP4. STRA6-deficient mice show a normal Vitamin A metabolism and various lines of evidence, including phenotypic, genetic, cellular, and signaling studies support the existence of unidentified holo-RBP4 receptors.^21,55^ Thus, it is conceivable that CD4⁺ T cells possess an as-yet-unidentified cell-surface receptor for holo-RBP4. Responsive and non-responsive J-Lat cells may provide means to uncover this receptor, e.g., by ligand-directed proteomics or unbiased CRISPR screening. Our data show that RBP4 activates latent HIV-1 via canonical NF-κB signaling. Usually, activation of NF-κB involves the upregulation of hundreds if not thousands of cellular genes. Thus, it is striking that RBP4 induces a very distinct cellular response involving upregulation of a very small set of genes. How this high specificity of RBP4 signaling for HIV-1 reactivation is achieved needs further investigation. This specificity also makes holo-RBP4 a promising candidate to explore potential synergistic effects with other LRAs.

In conclusion, our study shows that holo-RBP4 plays a previously unrecognized role in the activation of latent HIV-1 proviruses by mechanisms involving several signaling pathways but independently of RA production. These results add to the evidence that RBP4, retinol and its metabolites affect viral latency by a variety of non-exclusive mechanisms and further supports the existence of as-yet-unidentified holo-RBP4 receptors. As the primary carrier protein for retinol in the bloodstream, RBP4 delivers retinol to a wide range of tissues, such as the thymus, spleen, and lymph nodes. Thus, holo-RBP4 may exert effects in lymphoid tissues, the major site of the latent reservoirs of HIV. Our findings indicate that holo-RBP4 provides a new target to attack the latent reservoirs of HIV-1. Our results also demonstrate that the screening of peptide/proteins libraries from body fluids allows to discover effective natural LRAs. Currently, we are using this approach to identify endogenous agents reactivating HIV-1 proviruses even in latency models that are poorly responsive to PMA, TNF-α, and other known LRAs.

## Materials and methods

### Purification of active RBP4 from human hemofiltrate

Hemofiltrate is obtained during hemodialysis of patients with chronic renal failure. From 10,000 of liters of hemofiltrate (batch 050905) a peptide library was generated.^12^ Peptides were separated into 384 fractions based on charge (cation-exchange separation) and hydrophobicity (reversed-phase liquid chromatography). This hemofiltrate peptide library is a salt-free source of highly concentrated peptides and small proteins (<30 kDa).^13^ A J-Lat GFP reporter cell assay was carried out to identify HIV activating hemofiltrate fractions. One additional round of chromatographic purification in combination with the bioassay was performed to purify the active compound. For reversed-phase separation, the Pool 4 fraction was subjected to reversed-phase chromatography on Source RPC 15 (Cytiva, USA) of dimensions 2 × 25 cm at a flow rate of 13 ml/min, using a linear gradient of acetonitrile using aqueous buffer A (0.1% TFA) and B (80% acetonitrile, 0.05%TFA), resulting in bioactive fraction P4, frs. 27-38. Detection of eluting compounds was monitored at 280 nm. Using these bioactive fractions, the final purification step was performed on a Phenomenex Aqua RP18 (Phenomenex, USA) of dimensions 1 × 25 cm at a flow rate of 2.5 mL/min, using a linear gradient of acetonitrile using same RP buffers resulting in the main bioactive fractions 34 and 42. Fraction 42, of higher purity, was subjected to mass spectrometry and sequence analyses.

### Molecular mass measurement of RBP4 by MALDI-TOF MS

The sample was analyzed using an Axima Confidence MALDI-TOF MS (Shimadzu, Japan) in positive linear mode on a 384-spot stainless-steel sample plate. Spots were coated with 1 µL 5 mg/mL CHCA previously dissolved in matrix diluent (Shimadzu, Japan), and the solvent was allowed to air dry. Then, 0.5 µL sample or standard was applied onto the dry pre-coated well and immediately mixed with 0.5 µL matrix; the solvent was allowed to air dry. All spectra were acquired in the positive ion linear mode using a 337-nm N2 laser. Ions were accelerated from the source at 20 kV. A hundred profiles were acquired per sample, and 20 shots were accumulated per profile. Measurements and MS data processing were controlled by the MALDI-MS Application Shimadzu Biotech Launchpad 2.9.8.1 (Shimadzu, Japan).

### Cell culture

Latently HIV-1 infected cell lines were obtained from the NIH AIDS repository (J-Lat 6.3 #9846, J-Lat 8.4 #9847, J-Lat 9.2 #9848, J-Lat 10.6 #9849, J-Lat 15.4 #9850; A1 #9852, A7 #9853, A2 #9854, H2 #9855, Jurkat E6 J1.1 # 1340, U937 U1 #165, ACH-2 #ARP-349). Cells were cultivated in RPMI 1640 medium (Gibco) supplemented with 10% (v/v) FBS (Gibco), 100 U/ml penicillin (PAN-Biotech), 100 µg/ml streptomycin (PAN-Biotech), and 2 mM L-glutamine (PAN-Biotech). Cells were split 1:10 regularly 2–3 times a week.

### Generation and screening of HF-libraries

Fractions of a hemofiltrate-derived peptide library generated as described before^13^ were tested for their ability to reactivate latent HIV-1 proviruses in J-Lat 11.1 cells. For this, 100,000 J-Lat cells were seeded in F-bottomed 96-well dishes and incubated with the peptide fractions for 24 h. The next day, cells were analyzed for GFP expression by flow cytometry.

### Activity of purified RBP4 on several latently infected cell lines

To investigate the effect of purified RBP4 on latently infected cell lines, 300,000 cells were seeded in 48-well dishes and treated with plasma-derived, purified RBP4 (Medix Biochemica, #527-12-1) in duplicates. To examine the effect of RBP4 from different biological sources, RBP4 from human urine (Enzo Life Sciences) and recombinant human RBP4 (carrier-free) from Biolegend (Cat# 594906) were tested. As positive controls, cells were treated with 5 ng/mL PMA or TNFα. Two days post-treatment, cells were washed, fixed with 2% (v/v) PFA and analyzed by flow cytometry.

### Flow cytometry

All cells analyzed by flow cytometry were gated based on forward and side scatter characteristics, followed by exclusion of doublets, followed by (optional: the viability dye positive and negative cells and) the analysis of GFP (J-Lat full length and J-Lat GFP cells). In J1.1, U937 and ACH-2 cells, HIV reactivation was measured by p24 expression. To this purpose, cells were permeabilized 20 min with BD Cytofix/Cytoperm Fixation/Permeabilization Solution Kit (BD Biosciences). Cells were washed twice with 1X Perm/Wash solution, stained 30 min at RT with anti-HIV-1 p24 (RD1/KC57, Beckman Coulter #6604667, 1:100 in 1X Perm/Wash solution), and analyzed by flow cytometry after washing and fixing with 2% PFA. Data were generated with BD FACS Diva 6.1.3 Software using the FACS Canto II flow cytometer. Data analysis was performed using FlowJo 10.6 Software (Treestar) or FlowLogic Solution 1.0.

### SDS-PAGE

Hemofiltrate fractions or RBP4 from different sources were mixed with 6xSDS-PAGE loading buffer (187.5 mM Tris-HCl pH 6.8, 75% (v/v) Glycerol, 6% (w/v) SDS, 0.3% (w/v) Orange G, 15% (v/v) 2-mercaptoethanol, modified from LI-COR) and heated to 95 °C for 5 min. Samples were loaded on a precast 10% NuPAGE Bis-Tris gel (Invitrogen) and run for 2 h at 80 V in 1x MES SDS running buffer (Invitrogen). Subsequently, the gel was fixed with 50% MeOH/7% acetic acid for 15 min and washed 3 × 5 min with ultrapure water. Staining was performed with GelCode Blue Reagent (Thermo Fisher 24,590) overnight. The gel was de-stained using ultrapure water until the background appeared clear, and then imaged in a LiCor Odyssey system.

### RBP4 protein biochemistry

Deloading of holo-RBP4 (Medix Biochemica) to obtain retinol-free (apo-RBP4) was carried out as previously reported.^29^ In brief, holo-RBP4 was incubated overnight at 30°C with a 40:60 butanol:diisopropyl ether mixture to extract bound retinol. After centrifugation at 5000 rpm for 5 min, the aqueous phase containing apo-RBP4 was collected. This extraction was repeated six times with 12-h incubations in fresh solvent. Apo-RBP4 was then dialyzed for 6 h in 1× PBS using Membra-Cel™ tubing (Carl Roth; Cat. No. 1781.1) and the buffer was exchanged to water using Vivaspin® Turbo 15 concentrators (10 kDa MWCO; Sartorius). To regenerate holo-RBP4, apo-RBP4 was incubated with a 6-fold molar excess of retinol at 37 °C for 2.5 h. All steps used ultrapure water. Samples were kept on ice post-incubation, and ligand binding was assessed by fluorescence (excitation: 280 nm; emission: 340 nm) using a Tecan Spark microplate reader.

### Treatment with inhibitors

A total of 300,000 J-Lat cells were seeded in 48-well dishes and treated with serial dilutions of: 200, 400, 800 µM DEAB (4-Diethylaminobenzaldehyde, Cat# HY-W016645, MedChemExpress); 4, 8, 16 µM WIN 18446 (Bis-diamine, Cat# SML3187, Sigma Aldrich); 5, 10, 20, 40 µM JNK Inhibitor II (CAS 129-56-6, Calbiochem); 25, 50, 100 µM STAT5 inhibitor (Cat# Cay15784-5, Biomol); 50 µM Rottlerin (Rottlerin, Cat# R5648, Sigma-Aldrich); 50 nM Selumetinib (AZD6244, Cat# HY-50706, MedChemExpress); 50 µM U0126 (U0126, CAS 109511-58-2, Calbiochem); 2.5, 5, 10 µM of BAY 11-7082 (Cat#HY-13453, MedChemExpress); 1.5, 3, 6 µM NIK SM1 (Cat #HY-112433, MedChemExpress), or 2.5, 5, 10 µM B022 (Cat#HY-120501, MedChemExpress) and incubated for 4 h at 37 °C. Cells were then treated with 5 ng/mL of TNFα (Tumor Necrosis Factor-α human, Cat# H8916, Sigma-Aldrich), or different doses of RBP4 (Retinol Binding Protein 4, Cat#527-12-1, Medix Biochemica) and incubated at 37 °C. Forty-eight hours later, J-Lat cells were washed once with PBS and incubated with 100 µL of eBioscience™ Fixable Viability Dye eFluor™ 780 diluted 1:1000 (v/v) in PBS for 15 min at RT in the dark. Cell were washed twice in PBS, fixed in 4% PFA at 4 °C for 1 h and analyzed by flow cytometry.

### Treatment with retinoids

A total of 300,000 J-Lat cells were seeded in 48-well dishes and treated with serial dilutions of atRA (All-trans-Retinal, Cat# R2500, Sigma-Aldrich), RO (Retinol, Cat# R7632, Sigma-Aldrich), RA (Retinoic Acid, Cat# R2625, Sigma-Aldrich), 5 ng/mL of TNFα (Tumor Necrosis Factor-α human, Cat# H8916, Sigma-Aldrich), 100 µg/mL of Holo-RBP4 (Retinol Binding Protein 4, Cat#527-12-1, Medix Biochemica), 100 µg/mL of Apo-RBP4 (Recombinant Human RBP4 (carrier-free) Cat# 594906, Biolegend) and incubated at 37 °C. Forty-eight hours later, J-Lat cells were washed once with PBS and incubated with 100 µL of eBioscience™ Fixable Viability Dye eFluor™ 780 diluted 1:1000 (v/v) in PBS for 15 min at RT in the dark. Cells were washed twice in PBS, fixed in 4% PFA at 4 °C for 1 h and analyzed by flow cytometry.

### Treatment with LPS

A total of 150,000 J-Lat cells were seeded in 96-well plates and treated with 0.5 mg/mL of LPS (LPS-EK, Cat# 5973-44-01, Invivogen) in the highest concentration, diluted in a 1:10 titration row, and incubated at 37°C. Forty-eight hours later, the supernatants were collected for further analyses, and the J-Lat cells were washed once with PBS and incubated with 100 µL of eBioscience™ Fixable Viability Dye eFluor™ 780 diluted 1:1000 (v/v) in PBS for 15 min at RT in the dark. Cells were washed twice in PBS, fixed in 4% PFA at 4 °C for 1 h and analyzed by flow cytometry.

### Legendplex ELISA

The assay was performed according to the manufacturer’s instructions. In brief, supernatants of cells treated with RBP4 or LPS or left untreated were collected 48 h post-treatment and incubated for 2 h at room temperature with antibody-coated beads, followed by washing and incubation with the detection antibodies. Supernatants from MDMs treated with LPS were used as a positive control. After incubation with the staining reagent, the beads were fixed in PFA 2% and analyzed in a high-throughput sampler via flow cytometry (Cytoflex, Beckman). Quantification was performed using a standard and the Biolegend Legendplex v8.0 software.

### Assessment of CRISPR/Cas9 KO efficiency

A detailed description of how KO J-Lat cells were obtained is provided in the Supplementary Materials and Methods. Primers used to generate and verify the KOs are provided in Supplementary Tables 2 and 3, respectively. KO efficiencies (Supplementary Table 1) were quantified through the Tracking of Indels by DEcomposition software (https://apps.datacurators.nl/tide/) from Sanger sequencing chromatograms by comparing the NT control and the KO samples around the CRISPR/Cas9 cut site. As previously reported,^56^ TIDE first aligns the sgRNA sequence to the NT control sequence to determine the position of the expected Cas9 break site. Next, the signal intensities (A/T/C/G peak heights) from the KO sample are compared to the NT control sample, and any mixed/shifted peaks downstream of the cut site in the decomposition window are interpreted as aberrant nucleotides or indels. Subsequently, the TIDE software decomposes the composite sequence trace into its individual components and models the KO sequence as a mixture of control sequence and sequences with different indel sizes (−1 bp, −2 bp, +1 bp, etc.), and estimates the relative abundance of each indel (insertion and deletion) size in the total sequencing signal. The software provides the *R*^2^ value as a goodness-of-fit measure of the combination of the indels model, and calculates the statistical significance (*p* value) of detection for each indel by a two-tailed *t*-test of the variance–covariance matrix of the standard errors. The total KO efficiency is then calculated as the sum of the frequencies of all the significant indels in the decomposition.

### RNA-seq

A total of 300,000 J-Lat 10.6 and 8.4 cells were seeded in 48-well dishes and treated with 100 µg/mL of Holo-RBP4 (Retinol Binding Protein 4, Cat#527-12-1, Medix Biochemica) or left untreated, and incubated at 37 °C. Forty-eight hours later, J-Lat cells were washed once with PBS and RNA was harvested using QuickRNA Miniprep kit (Cat#R1055, Zymo) according to the manufacturer’s instructions. A total of 1000 ng of total RNA was used for the construction of sequencing libraries. RNA libraries for RNA-seq were prepared using the sparQ RNA-Seq HMR Kit (QuantaBio) with sparQ PureMag Beads (QuantaBio) and sparQ UDI Adapters (QuantaBio) following the manufacturer’s protocols. For data analysis, the proviral HIV-1 sequence from J-Lat10.6 (GenBank: MN989412.1) was added as a separate chromosome to hg38. Reads were quality checked with fastqc-0.11.8,^57^ mapped using STAR-2.7.9 aligner to this artificial genome^58^ and counted with featureCounts (subread-2.0.2).^59^ Differential gene expression analysis was performed with DESeq2 (v1.42.1),^60^ enrichment analysis searching the MSigDB Hallmark database^61^ was performed using enrichR (v 3.4).^62^

### Primary CD4+T cells

CD4+ T cells were isolated from buffy coats using the RosetteSep™ Human T Cell Enrichment Cocktail (Stem Cell, #15021) according to the manufacturer’s recommendation. CD4+ T cells were activated using Dynabeads Human T-Activator CD3/CD28 beads (Thermo Fisher), and cultured in RPMI-1640 medium containing 10% FCS, glutamine (2 mM), streptomycin (100 µg/ml), penicillin (100 U/ml) and interleukin 2 (IL-2, Miltenyi Biotec #130-097-745) (10 ng/ml).

### qRT-PCR

STRA6, TLR4, IL-6, and IL-1β mRNA levels were determined in cells and normalized to GAPDH expression levels. Total RNA was isolated using the RNeasy Plus Mini kit (Qiagen) according to the manufacturer’s instructions. qRT-PCR was performed according to the manufacturer’s instructions using TaqMan Fast Virus 1-Step Master Mix (Thermo Fisher) and a OneStepPlus Real-Time PCR System (96-well format, fast mode). All reactions were run in duplicates using TaqMan primers/probes from Thermo Fisher: GAPDH (Cat# 4310884E), STRA6 (Hs00980261_g1), IL-1β (Hs01555410_m1), IL-6 (Hs00174131_m1), TLR4 (Hs00152939_m1).

### RBP4 ELISA

Concentration of RBP4 in plasma was measured using Human RBP4 ELISA Kit (Cat# DRB400, R&D) according to manufacturer’s instructions.

### HIV-1 Gag-p24 single-molecule assay (Simoa)

Cryopreserved PBMCs from seven HIV-infected ART-suppressed study participants were used to generate ex vivo measures of HIV-1 latency reversal as quantified by HIV-1 gag-p24 Single-Molecule Assay (Simoa).^45,63^ Briefly, 20 million cells were cultured in 2 mL R10+ media plus 100 U/mL IL-2, 200 nM Raltegravir, in the presence of the test agent (RBP4) or anti-CD3/28 positive control and incubated for 72 h at 37 °C and 5% CO_2._ By way of trypan blue stain, live cells were counted after incubation. Cell pellets and culture supernatants were harvested for viral gag-p24 protein Simoa. To harvest cells pellets for Simoa, we resuspended pellets in Simoa buffer (1x protease inhibitor cocktail, 49% FBS, 1% Triton, 49% Blocker Casein in PBS (Thermo Fisher)) and incubated for 30 min and frozen at −80 °C. To harvest the culture supernatants, we added 10% Triton (in PBS) to the supernatants to a final concentration of 1% and stored them at −80°. The samples were sent to the University of Pennsylvania Human Immunology Core Facility and analyzed on the Quanterix HD-X machine using HIV p24 Quanterix kit (catalog 102215, lot 504201) according to the manufacturer protocol. Briefly, 278 µl of each sample and reagents were loaded into the plate and machine. The assay report was exported from the HD-X, and the results were reported as pg/mL.

### Intact proviral DNA assay (IPDA)

IPDA was used to assess the composition and levels of intact and defective proviruses in CD4+ T cells isolated from leukapheresis-derived PBMC as described.^43^ Accelevir Diagnostics performed sample processing and IPDA quantification in accordance with the company standard guidelines by blinded operators. Briefly, isolation of the CD4+ T cells from cryopreserved PBMCs was conducted using the EasySep Human CD4+ T cell Enrichment Kit (Stemcell Technologies). QIAamp DNA Mini Kit (Qiagen) was used to extract genomic DNA, whose concentration was then measured using fluorometry (Qubit dsDNA BR Assay Kit, Thermo Fisher Scientific). The quality was assessed using ultraviolet-visible (UV/VIS) spectrophotometry (QIAxpert, Qiagen). Finally, the genomic DNA was assessed by IPDA using the Bio-Rad QX200 AutoDG Droplet Digital PCR system. Results were expressed as count of total proviruses, intact, 3’ or 5’ deleted and/or hypermutated HIV pro-viruses per 106 CD4+ T cells.

### OpenAI

ChatGPT was used to optimize language and streamline the manuscript.

### Statistical analysis

Results were graphed by Graph Pad Prism 10, and are displayed as means ± standard error of the mean (SEM). Unless otherwise stated, *p* values were determined using a two-tailed Student’s *t* test with Welch’s correction or by one-way ANOVA. Linear correlations and statistics were performed using GraphPad Prism 10 simple linear regression. Reactivation of HIV in cells from PLWH was determined using the Wilcoxon Signed-Rank test. *p* values ≤ 0.05 were considered statistically significant. A *p* value below 0.05 is annotated with *, *p* values below 0.01 with **, and *p* values below 0.001 with ***.

## Supplementary information

Uncropped SDS PAGE

Supplementary Materials

## Data Availability

All data are available in the main text or the Supplementary Materials. Materials are available from the corresponding author. Primary data are available at https://data.mendeley.com/datasets/wp9fht9k3k/2. RNA sequencing data have been submitted to the GEO repository and have been assigned accession numbers GSM9110570 to GSM9110581.
