# Supplementary figures and images for: Retinol Binding Protein 4 reactivates latent HIV-1 by triggering canonical NF-κB, JAK/STAT5 and JNK signalling
Source: Signal Transduct Target Ther. 2025 Oct 3;10:326. doi: 10.1038/s41392-025-02424-3 (PMC12491451; doi:10.1038/s41392-025-02424-3)

Uncropped SDS-PAGE Figures

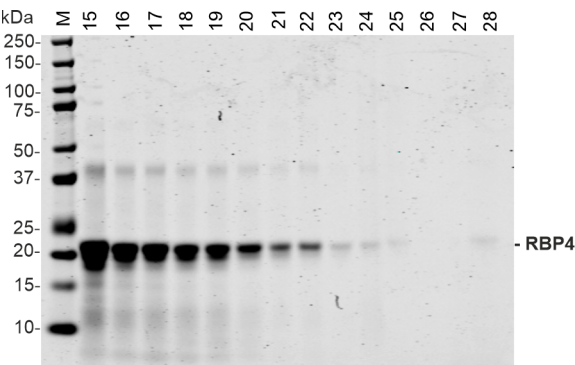

Figure 1c

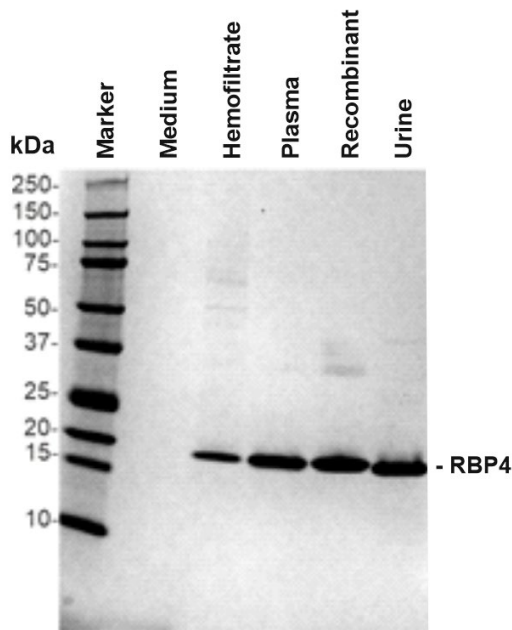

Figure 3b

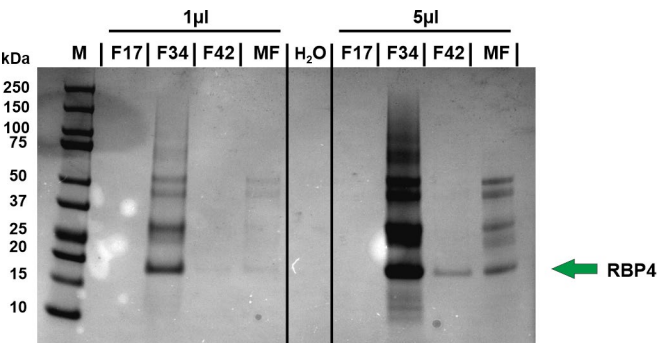

Figure S1b

Supplement: Supplementary file 1 — Uncropped SDS PAGE [file 41392_2025_2424_MOESM1_ESM.pdf]
