# Supplementary material for: Retinol Binding Protein 4 reactivates latent HIV-1 by triggering canonical NF-κB, JAK/STAT5 and JNK signalling
Source: Signal Transduct Target Ther. 2025 Oct 3;10:326. doi: 10.1038/s41392-025-02424-3 (PMC12491451; doi:10.1038/s41392-025-02424-3)
Supplement: Supplementary file 2 — Supplementary Materials [file 41392_2025_2424_MOESM2_ESM.docx]

Supplementary Materials for

Retinol Binding Protein 4 reactivates latent HIV-1
by triggering canonical NF-κB, JAK/STAT5 and JNK signalling

Chiara Pastorio^1^, Khumoekae Richard^2^, Shariq Usmani^1^, Ann-Kathrin Kissmann^3^, Grigory Bolotnikov^3^, Guillermo Gosálbez^1^, Manuel Hayn^1^, Lennart Koepke^1^, Andrea Preising^1^, Alina Sauertnik^1^, Nico Preising^4^, Ludger Ständker^4^, Matthew Fair^2^, Jessicamarie Morris^2^, Emmanouil Papasavvas^2^, Qin Liu^2^, Honghong Sun^5^, Armando Rodríguez^4,6^, Karam Mounzer^2^, Sebastian Wiese^4,6^, Pablo Tebas^5^, Yangzhu Du^5^, Gregory M. Laird^7^, Markus Jaritz^8^, Frank Rosenau^3^, Moritz M. Gaidt^8^, Konstantin M.J. Sparrer^1,9^, Luis J. Montaner^2^, Frank Kirchhoff^1^

Correspondence to: frank.kirchhoff@uni-ulm.de

**This PDF file includes:**

Materials and Methods

Figures. S1 to S14

Tables S1 to S3

Uncropped SDS-Page images

Material and Methods

**Identification of RBP4 by LC-MS/MS sequencing.**

The sample was reduced with 5 mM DTT for 20 min at RT, then carbamidomethylated with 50 mM iodoacetamide for 20 min at 37 °C, and subsequently digested with trypsin (ThermoFisher Scientific, 900,589), at a 1:50 ratio (enzyme: protein) for 16 h at 37 °C. A 15 µl aliquot of the digested sample was analyzed using an Orbitrap Elite Hybrid mass spectrometry system (Thermo Fisher Scientific) online coupled to an U3000 RSLCnano (Thermo Fisher Scientific) uPLC as described ^62^. XCalibur 2.2 SP1.48 (Thermo Fisher Scientific, Bremen, Germany) was used for data-dependent tandem mass spectrometry (MS/MS) analyses. Database search (PEAKS DB) was performed using PEAKs X + studio. For peptide identification, MS/MS spectra were correlated with the UniProt human reference proteome set (Uniprot release 2023_03; 20,423 reviewed entries). Parent mass error tolerance and fragment mass error tolerance were set at 15 ppm and 0.5 Da, respectively. The maximal number of missed cleavages was set at 3. Carbamidomethylated cysteine was considered as a fixed modification, and methionine oxidation as a variable modification. False discovery rates were set on the peptide level to 1%.

**RBP4 rGO-FET-based Biosensing.**

To functionalize the rGO-FETs, a solution was prepared by dissolving 500 μM PyPEG (a polyethylene glycol derivative featuring a PBSE linker) and 50 μM 1-pyrenecarboxylic acid (PCA) in DMSO. The gFET devices were submerged in this mixture and incubated in the dark at ambient temperature for 24 hours. Following incubation, the chips were rinsed thrice with 1 mL of isopropanol and dried under a gentle stream of nitrogen gas. To assess biosensing capabilities, the transfer characteristics (IDSVG) of the gFETs were recorded using a Keysight U2722A source/measure unit (Keysight Technologies, Santa Rosa, CA, USA) in combination with a custom LabView interface (National Instruments, Austin, TX, USA). Measurements were conducted across a gate voltage sweep from –0.5 V to +0.5 V at a scan rate of 20 mV/s, with the drain-source voltage (VDS) fixed at 50 mV. Before aptamer coupling, the chip surface was flushed with distilled water at 0.2 mL/min for 10 minutes, followed by 0.01× DPBS at 0.5 mL/min for 2 minutes. After these washing steps, baseline current–voltage characteristics were recorded. For surface activation, a freshly prepared 0.01× DPBS solution containing 15 mM EDC and 15 mM NHS (1 mL) was introduced to the gFETs at 0.2 mL/min for 30 minutes to activate carboxyl functionalities. The system was then flushed with 0.01× DPBS at 0.5 mL/min for 1 minute, and subsequently at 0.2 mL/min for 10 minutes to remove residual crosslinking agents. Next, 1 mL of 1× DPBS containing 100 pmol of amino-modified aptamer library was circulated across the chip at 0.2 mL/min for 1 hour to enable covalent attachment of the aptamer to the surface. Following functionalization, another IDSVG scan was performed to confirm successful immobilization of the aptamer. Samples were measured by Injecting 1 mL of protein solution (3 nM) at a flowrate of 0.5 mL/min for 2 minutes, before incubating the analyte for 10 minutes at 0.2 mL/min. After incubation the chips were rinsed with 1X DPBS for 5 minutes. Subsequently an ID-VG curve was recorded. Data analysis and graph generation were performed using OriginPro 2022b (OriginLab Corporation, Northampton, MA, USA).

**CRISPR/Cas9 KO in J-Lat cells.**

1 x 10^6^ J-Lat 10.6 cells were transfected with the HiFi Cas9 Nuclease V3 (IDT)/gRNA complex (80pmol/500pmol) (Lonza) using specific sgRNAs or NT control (Supplementary Table 2), using the Amaxa SE Cell Line 4D-Nucleofector® X Kit (Lonza, Cat#V4XC-1032), pulse code CL-120. At four days post Cas9/sgRNA-transfection, 1 x 10^6^ J-Lat 10.6 cells/sample were used for genomic DNA isolation using the QIAamp DNA Mini Kit (Qiagen, Cat#51306) according to the manufacturer’s instructions. The target sequence was amplified through PCR using specific forward and reverse primers (Biomers) (Supplementary Table 3). PCR products were purified using the Monarch® Spin PCR & DNA Cleanup Kit (NEB, Cat# T1130L). The target genes KO efficiency was verified through sanger sequencing compared to the NT control using Tracking of Indels by Decomposition software (https://apps.datacurators.nl/tide/). Based on the quantitative sequence trace data from the NT control and the target gene sequencing reactions, the TIDE software allows to assess and quantify the CRISPR/Cas9 KO efficacy (Supplementary Table 1), and identifies the predominant types of insertions and deletions (indels) in the target sample. The same day, 150,000 cells/sample were treated with 20, 40 µM of JNK inhibitor (JNK Inhibitor II, CAS 129-56-6, Calbiochem) or 50, 100 µM of STAT5 inhibitor (STAT5 Inhibitor, Cat# Cay15784-5, Biomol) or both or left untreated, and incubated for 4 hs at 37°C. Cells were then treated with 5 ng/mL of TNFα (Tumor Necrosis Factor-α human, Cat# H8916, Sigma-Aldrich), 6, 12 or 25 µg/mL of RBP4 (Retinol Binding Protein 4, Cat#527-12-1, Medix Biochemica) and incubated at 37°C. 48 hs later, J-Lat cells were washed once with PBS and incubated with 100µL of eBioscience™ Fixable Viability Dye eFluor™ 780 diluted 1:1,000 (v/v) in PBS for 15 minutes at RT in the dark. Cell were washed twice in PBS, fixed in 4% PFA at 4°C for 1 h and analysed by flow cytometry.

**
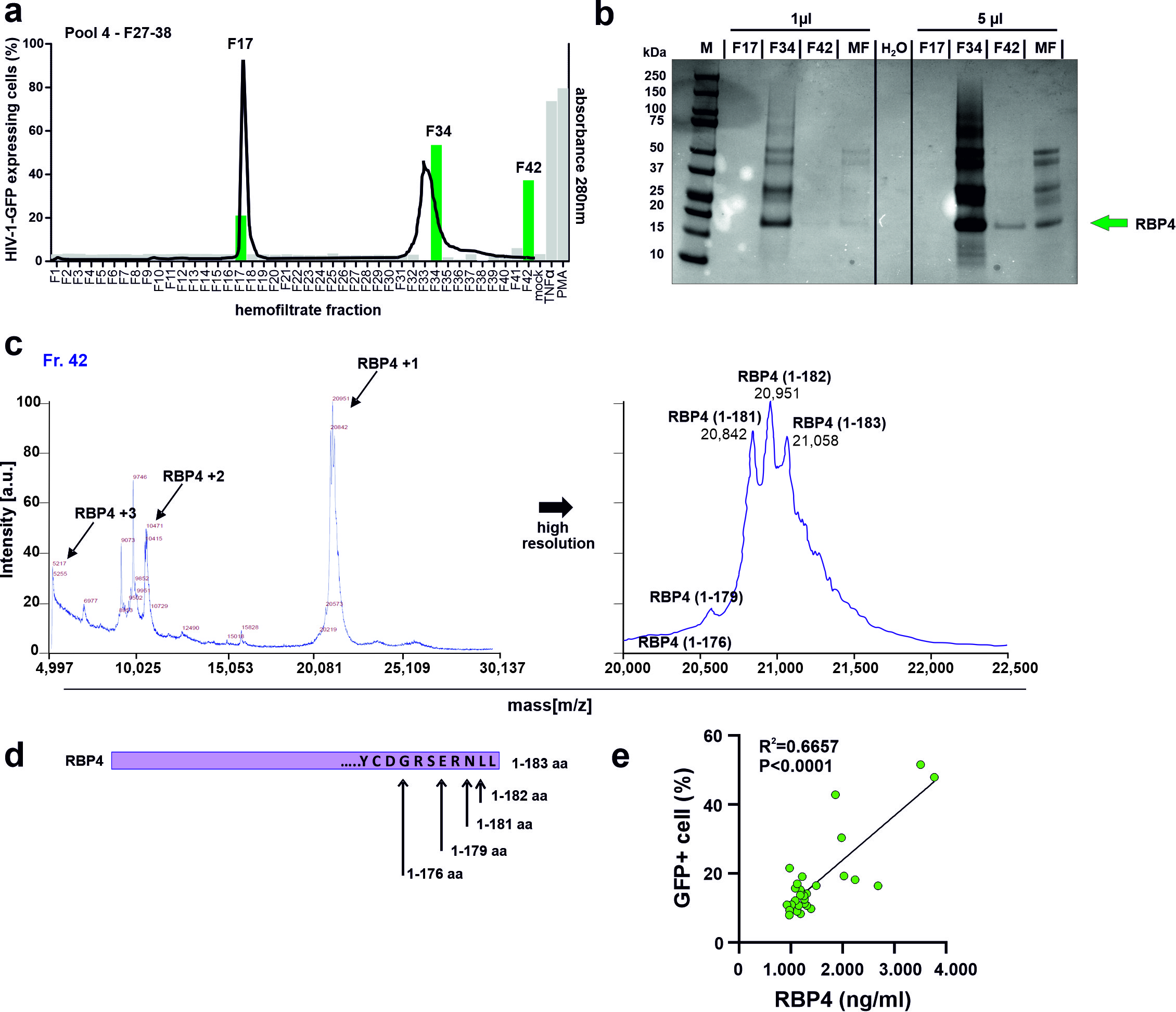
Figure. S1.**

**Identification of RBP4 as activator of latent HIV-proviruses**. (**a**) Bars indicate the percentage of activated (eGFP positive) J-Lat 11.1 cells in presence of the peptide fractions from pool 4 of the hemofiltrate-derived library, showed in Figure 1a. The black line indicates the peptide/protein elution profile. Green bars indicate the fractions used for further purifications. Mock indicates absence of the peptide fractions. PMA and TNFα are used as positive controls. (**b**) Coomassie Brilliant Blue staining of a blot of the green HF-derived fractions from (a). The green arrow indicates the presence of a protein of ~21 kDa in active fractions. Fraction 42 was used for MS analysis since fraction 34 still contained a variety of hemofiltrate peptides and proteins. MF: mother fraction. (**c**) Identification of RBP4. The protein was carbamidomethylated, digested with Trypsin, and analyzed with a nanoLC-Orbitrap Elite system. Analysis of the proteolytic fragments showed the presence of RBP4, with a sequence coverage of 91.7% for the full-length mature RBP4 precursor. The MALDI-TOF spectrum of RBP4 shows a dominant signal of m/z 20960.75, which closely matches the theoretical m/z value of the RPB4 (1-182, 20959.42 Da) variant lacking the C-terminal leucin. (**d**) Depiction of RBP4 forms with C-terminal truncations. (**e**) Correlation between concentration of RBP4 in the active HR-derived fractions and the percentage of activated (eGFP+) J-Lat 11.1 cells. Coefficient of determination (R^2^ -values) and two tailed p value are provided.


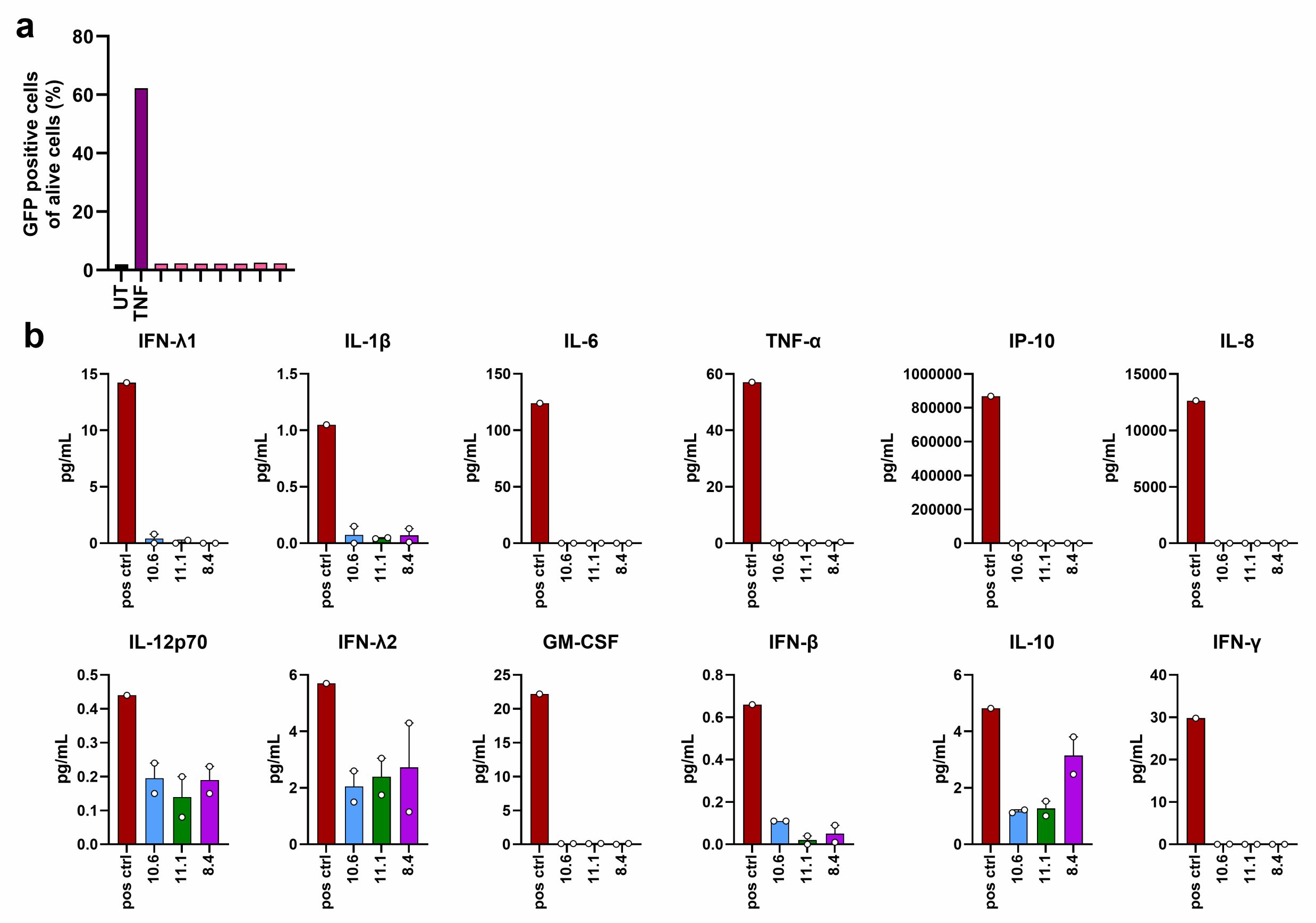


**Figure. S2.**

**Impact of LPS on HIV reactivation and cytokine expression in J-Lat cells.** (**a**) J-Lat 10.6 cells were left untreated or treated with 0.5 mg/mL of LPS in the highest concentration and diluted in a 1:10 titration row. TNF-α is shown for comparison. The percentages of GFP+ cells were measured by flow cytometry. (**b**) Absolute levels of the indicated cytokines in the supernatants of J-Lat 10.6, 11.1 or 8.4 cells collected 48 hours after treatment with 5 µg/mL of LPS. Supernatants from MDMs treated with LPS represent the positive control. Data are represented as mean ± SEM of two independent experiments.


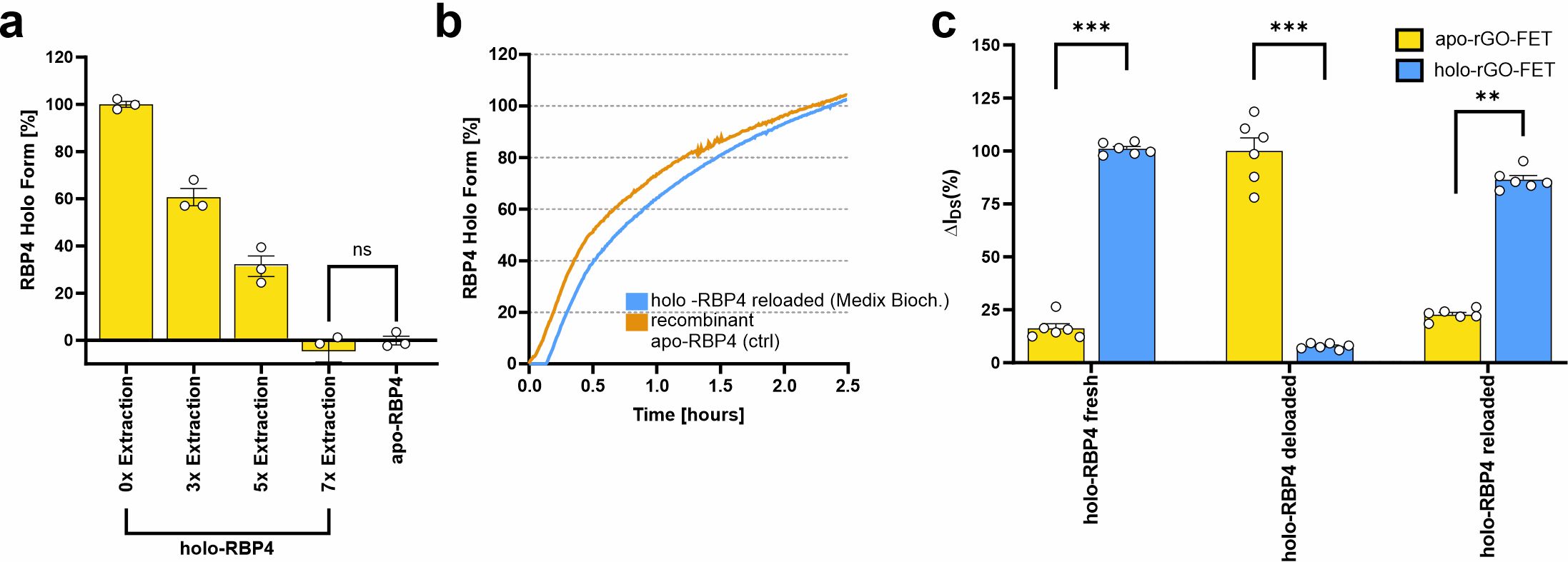


**Figure. S3.**

**Distinction between apo- and holo-RBP4 based on intrinsic fluorescence.** (**a**) Extraction of bound retinol from holo-RBP4 using 40% n-butanol/60% di-isopropyl ether (v/v), leading to progressive transition to the apo form (Ex: 280 nm/Em: 340 nm). Data are presented as mean ± SEM of six independent experiments. (**b**) Kinetics of retinol loading into apo-RBP4 measured by intrinsic fluorescence (Ex: 280 nm/Em: 340 nm). (**c**) Label-free discrimination of apo- and holo-RBP4 by two independent reduced graphene oxide field-effect transistors (rGO-FETs) functionalized with specific apo-/holo- aptamer libraries. Deloaded (apo) RBP4 was reloaded with retinol and compared to apo- and holo-RBP4 controls. Apo- and holo-RBP4 were flowed over two independent rGO-FET chips, showing distinct electrical responses corresponding to their respective conformational states. Data are presented as mean ± SEM of six independent experiments. P-values are shown as measured by two-tailed unpaired Student’s t test.


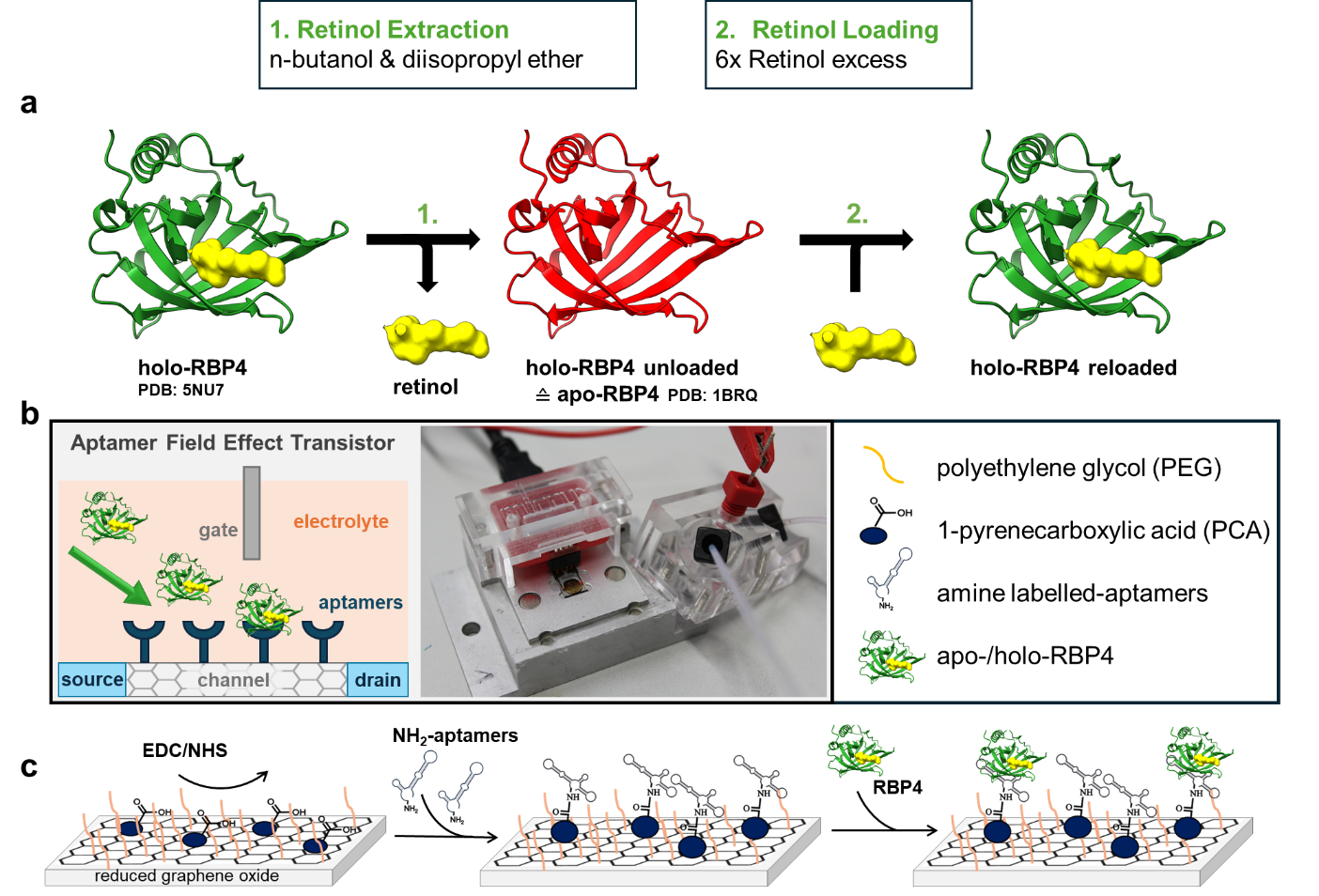
**Figure. S4.**

**Overview of retinol extraction/loading, biosensor setup, and aptamer immobilization for apo-/holo-RBP4 detection using rGO-FETs.** (**a**) Retinol is extracted from holo-RBP4 (PDB: 5NU7) using a n-butanol/diisopropyl ether mixture to yield apo-RBP4 (PDB: 1BRQ) (red). Reloading with a sixfold excess of retinol regenerates holo-RBP4 (green). (**b**) Illustration and photograph of the aptamer-functionalized field-effect transistor (FET) biosensor setup. The sensor detects conformationally distinct RBP4 isoforms via aptamer-mediated recognition at the gate-electrolyte interface. (**c**) Stepwise functionalization of the reduced graphene oxide (rGO) channel with carboxylic acid-containing linkers, amino-modified aptamers, and subsequent RBP4 binding.


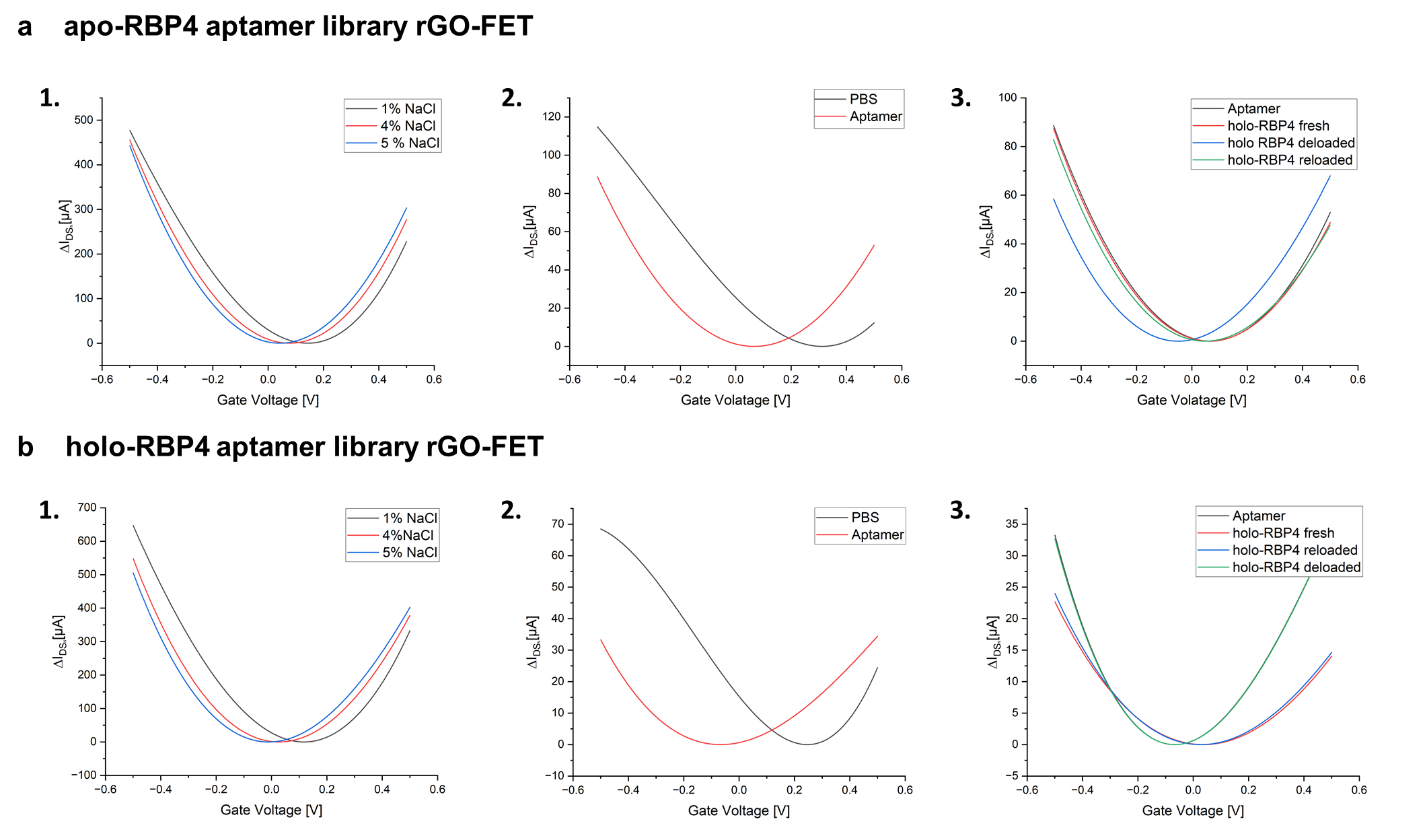
**Figure. S5.**

**I_DS_V_G_ characterization of rGO-FET biosensors obtained using sweeping the gate voltage from -0.5 V to 0.5 V** using (**a**) apo-RBP4 and (**b**) holo-RBP4 specific polyclonal aptamer libraries. 1. Device response during calibration of rGO-FET biosensors with increasing NaCl concentrations. Source-drain current was recorded to assess the sensitivity of the device to ionic strength variations. 2. Functionalization of reduced graphene surfaces using PyPEG linkers and subsequently EDC/NHS-mediated coupling of NH_2_-labeled apo-RBP4-Aptamer-library and holo-RBP4-Aptamer-library. 3. Binding apo-RBP4-Aptamer-library and holo-RBP4-Aptamer-library to holo-RBP4 samples. I_DS_V_G_ curves represent mean values of measurements conducted in triplicates.


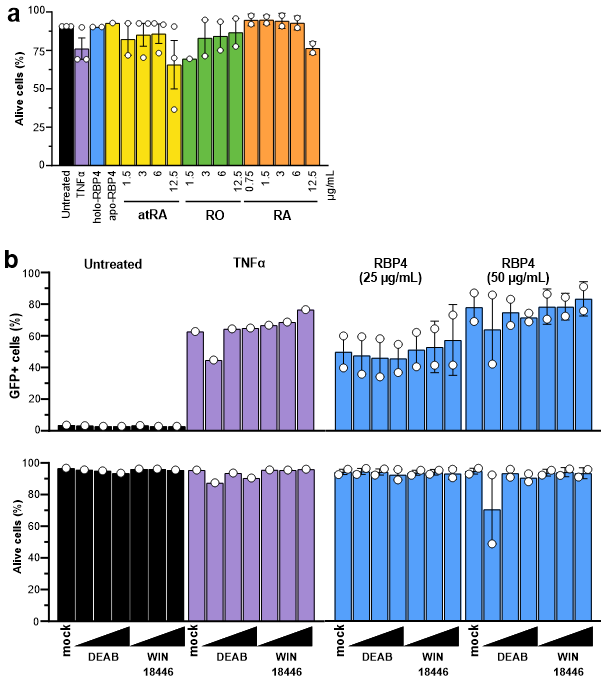


**Figure. S6.**

**Impact of RBP4 and free retinol and its metabolic products on HIV reactivation and cell viability.** (**a**) J-Lat 10.6 cells were treated with the indicated concentrations of all trans-retinal (atRA), retinol (RO) and retinoic acid (RA) or with TNFα, holo-RBP4 or apo-RBP4 and the percentage of alive cells were determined by flow cytometry. Data are represented as mean ± SEM of two independent experiments.(**b**) J-Lat 10.6 cells were pre-treated with DEAB or WIN18,446 and subsequently left untreated or treated with TNFα or the indicated concentrations of RBP4. The percentages of GFP+ (upper) and alive (lower) cells were measured by flow cytometry. Data are represented as mean ± SEM of two independent experiments.

**
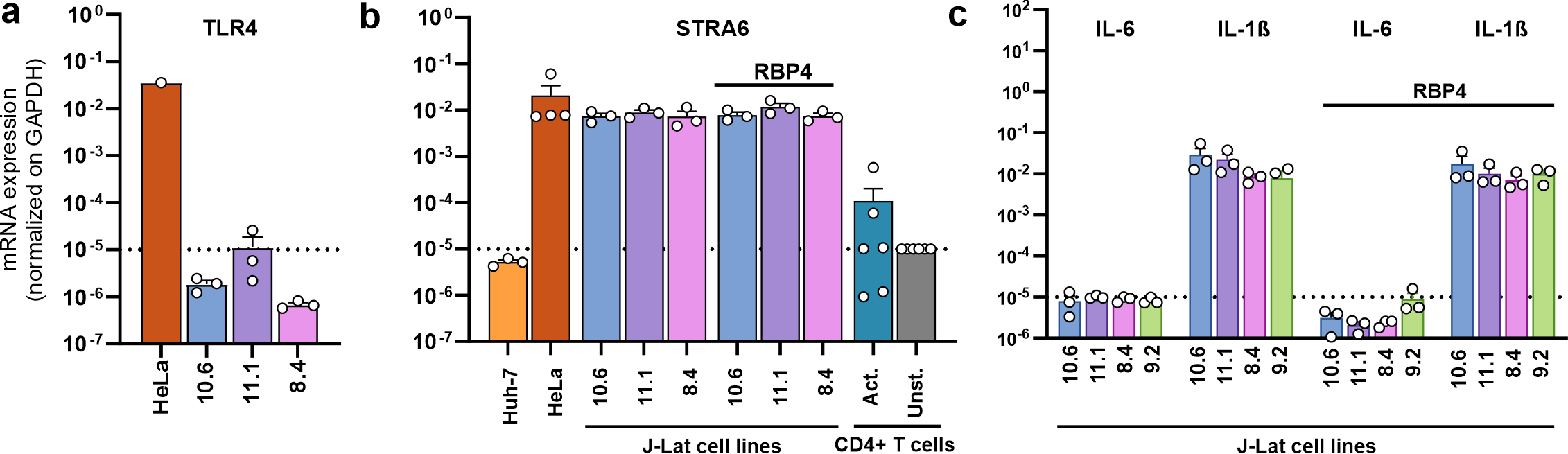
**

**Figure. S7.**

**TLR4, STRA6, IL-6 and IL-1β mRNA expression in J-Lat cells.** Levels of TLR4 (**a**), STRA6 (**b**), IL-6 and IL-1β (**c**) mRNA expression in the indicated J-Lat cell lines that were left untreated or treated with 50 µg/mL of RBP4 or in primary CD4+ unstimulated or activated T cells. HeLa and Huh-7 are shown for comparison. mRNA levels of the target genes were normalized on the GAPDH levels. Data are represented as mean ± SEM of three independent experiments.


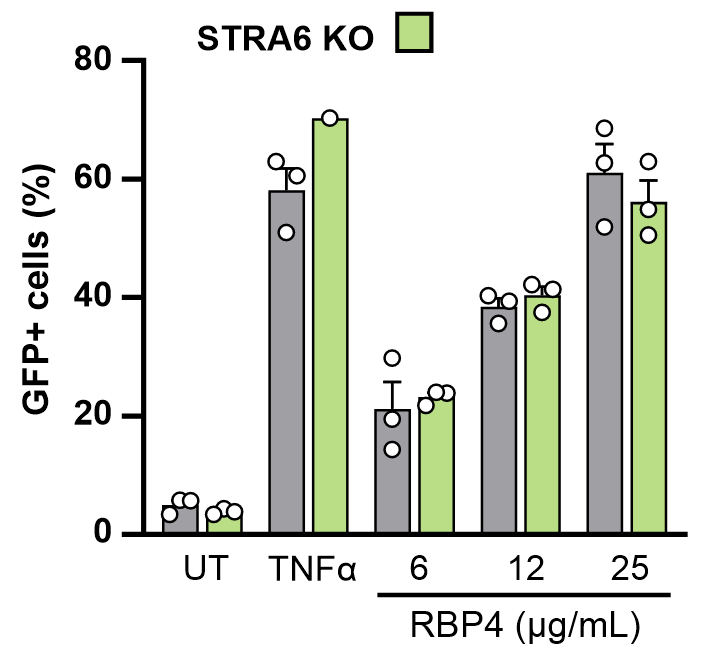


**Figure. S8.**

**Impact of STRA6 KO in RBP4-mediated HIV reactivation.** Reactivation of HIV-1 eGFP reporter proviruses in NT control (grey) STRA6 (green) KO J-Lat 10.6 cells treated with the indicated concentrations or holo-RBP4, TNFα or left untreated. The percentage of cells showing reactivation of HIV-1 eGFP reporter proviruses were determined by flow cytometry. Data are represented as mean ± SEM of three independent experiments.

**
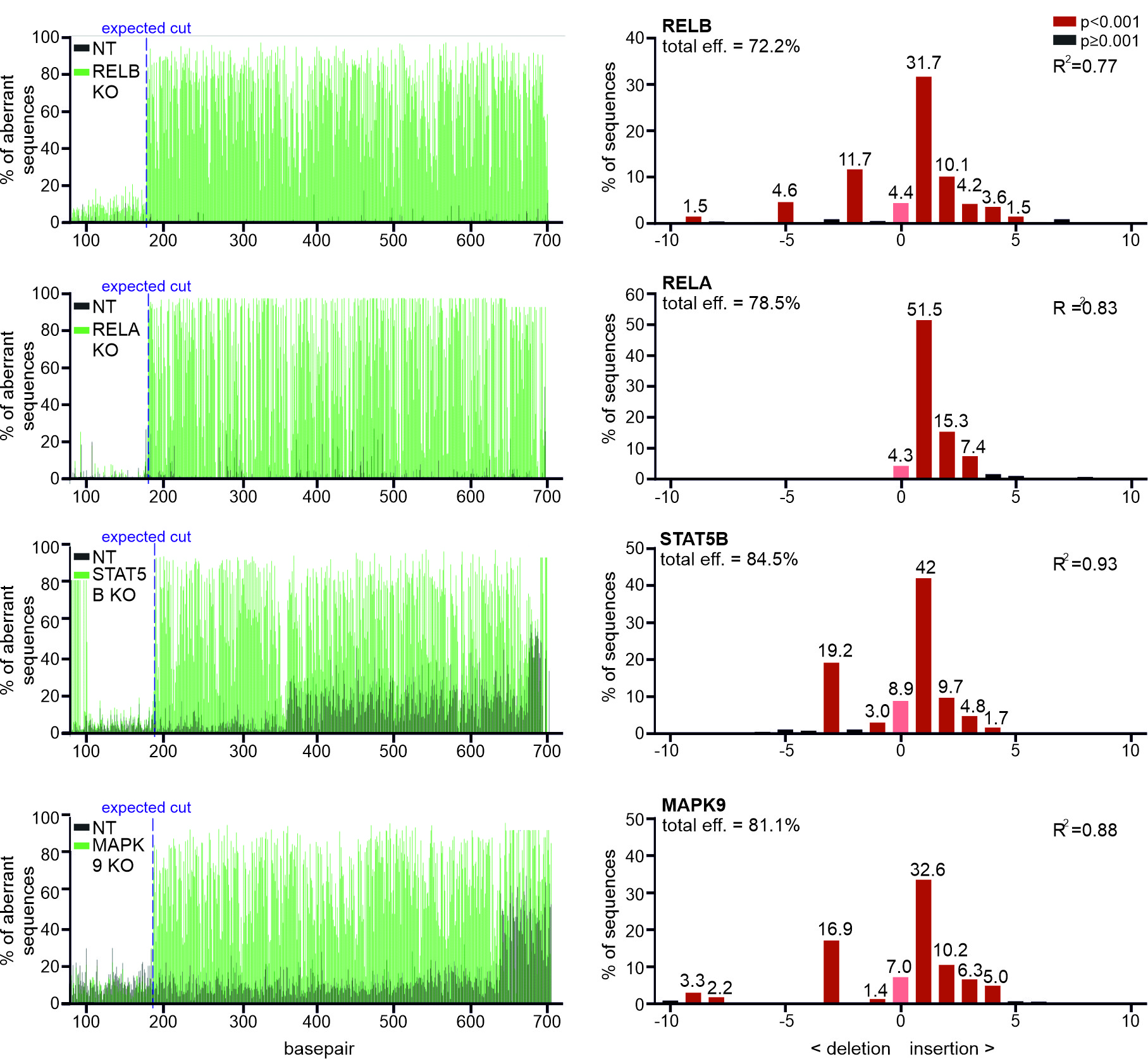
Figure. S9.**

**Assessment of CRISPR/Cas9 KO efficiencies in J-Lat 10.6 cells by sequence trace decomposition.** The left part shows representative plots of the aberrant sequence signal in the Non-Targeting (NT) control (black) and KO sample of the indicated genes (green) in J-Lat 10.6 cells determined by TIDE. The expected break site is indicated with a vertical dotted blue line and the region afterwards is used for decomposition. The bar diagrams on the right show the spectrum of indels obtained from the decomposition plots of each target gene for one representative KO experiment. The X-axis indicates the nature of indels, while the Y-axis depicts their relative abundance, which is also indicated by the numbers above the columns. The R^2^ value is a goodness-of-fit measure of the combination of indels model and the P-value shows the significance of detection of each indel.


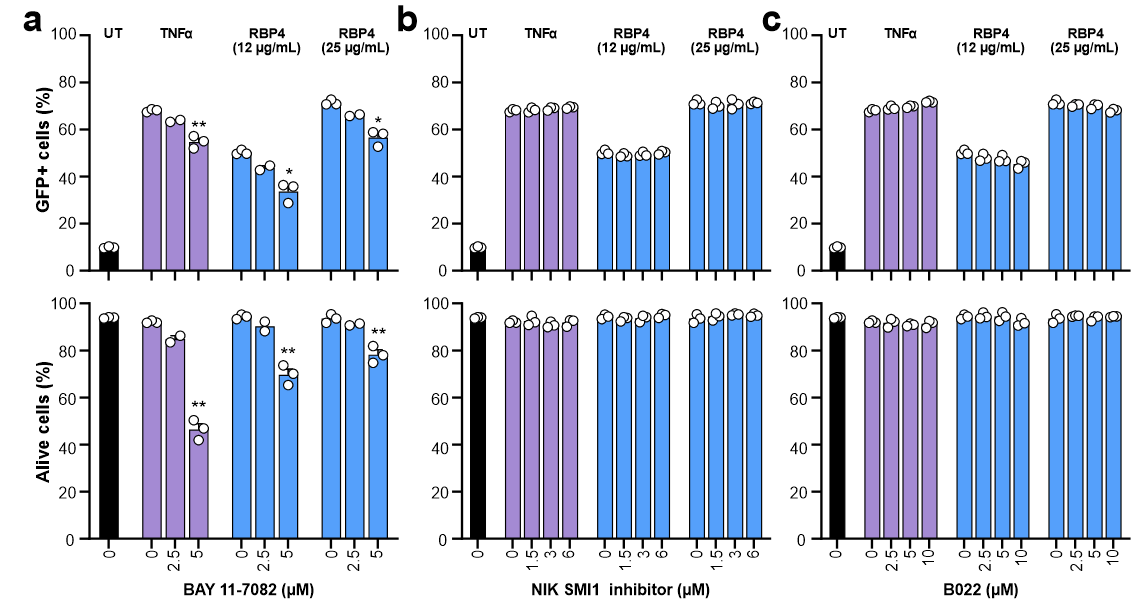


**Figure. S10.**

**Role of NF-κB signalling in RBP4-mediated HIV reactivation.** **(a-c**) J-Lat 10.6 cells were pre-treated with the indicated concentrations of the canonical NF-kB inhibitor BAY-11-7082 (**a**) or the non-canonical NF-kB inhibitors NIK SMI1 (**b**) or B022 (**c**). 4 hours after, the cells were left untreated or treated with TNFα or the indicated concentrations of RBP4. The percentage of cells showing reactivation of HIV-1 eGFP reporter proviruses (upper panel) and the cell viability (lower panel) were determined by flow cytometry. Data are represented as mean ± SEM of three independent experiments. P-values are shown as measured by two-tailed Student’s t test with Welch’s correction.


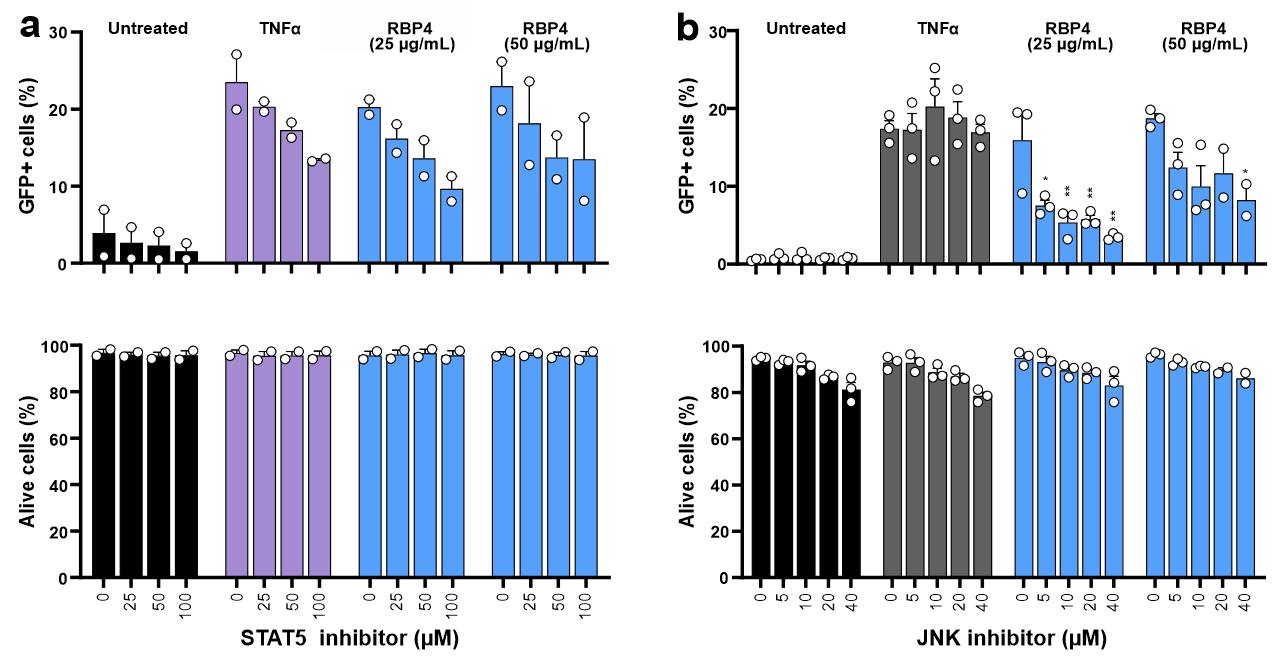


**Figure. S11.**

**Role of Jak/STAT and TLR/JNK activation in RBP4-mediated HIV reactivation.** (**a**)-(**b**) J-Lat 11.1 cells were pre-treated with the indicated concentrations of STAT5 (a) or JNK (b) inhibitor. 4 hours after, the cells were left untreated or treated with TNFα or the indicated concentrations of RBP4. The percentage of cells showing reactivation of HIV-1 eGFP reporter proviruses (upper panel) and the cell viability (lower panel) were determined by flow cytometry. Data are represented as mean ± SEM of two-three independent experiments. P-value are shown as measured by one-way ANOVA.


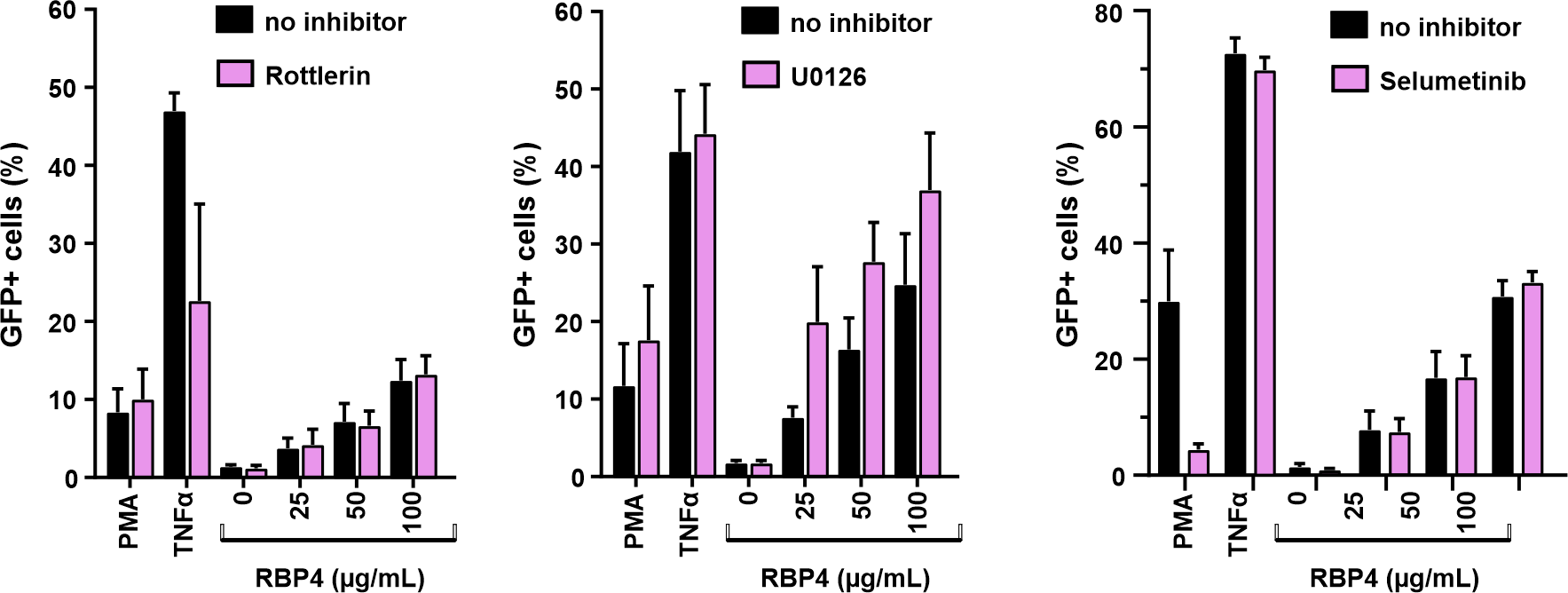


**Figure. S12.**

**Impact of PKC, MAPK and MEK1/2 on RBP4-mediated HIV reactivation**. J-Lat 10.6 cells were left untreated (black bars) or pre-treated (pink bars) with the indicated concentrations of the PKC inhibitor Rottlerin (left), the ERK1/2 inhibitor UO126 (middle), or the MEK1/2 inhibitor Seleumetinib (right). Four hs later, the cells were treated with PMA, TNFα or the indicated concentrations of RBP4. The percentage of cells showing reactivation of HIV-1 eGFP reporter proviruses were determined by flow cytometry. Data are represented as mean ± SEM of three-five independent experiments.


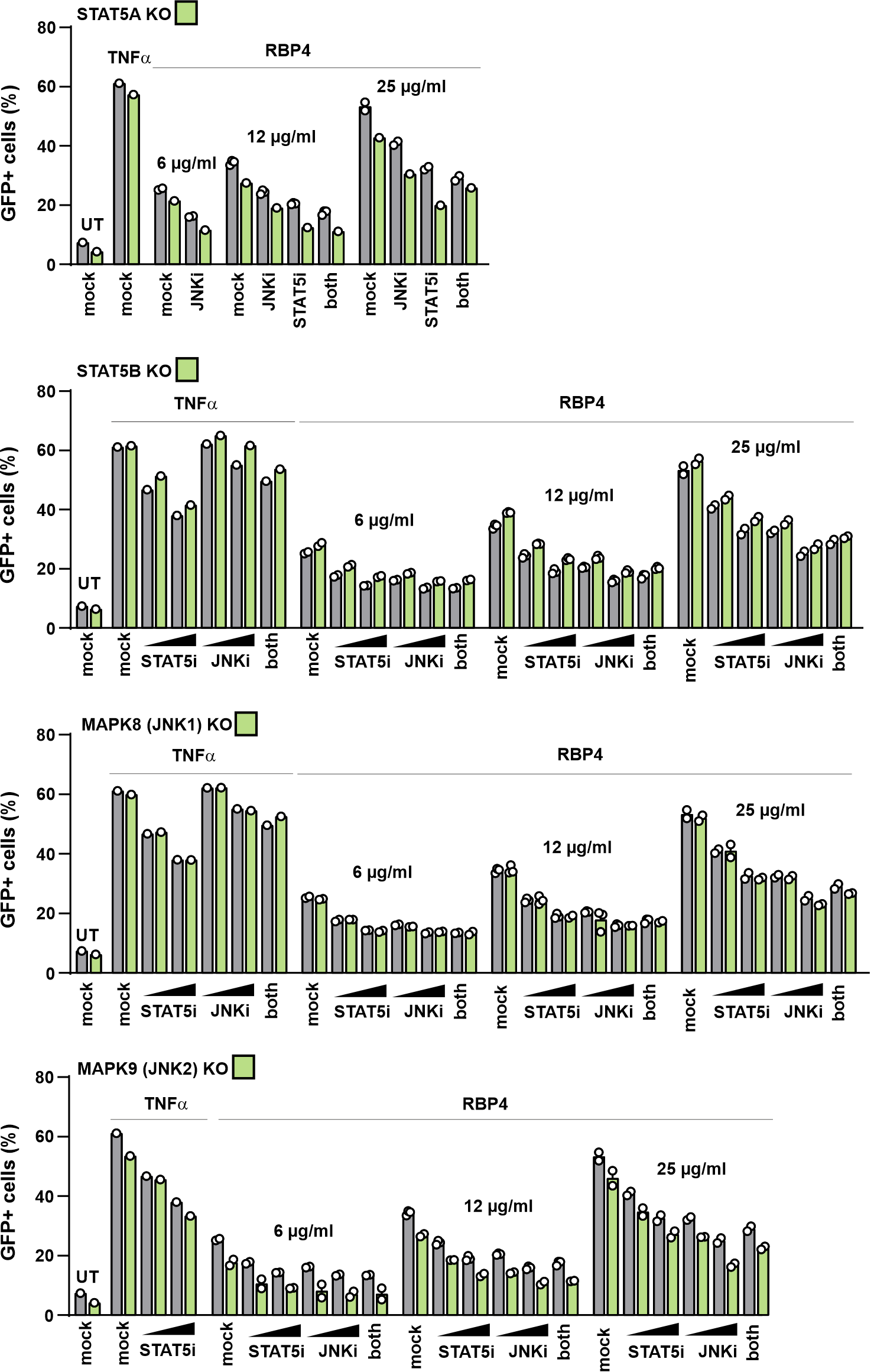


**Figure. S13.**

**RBP4-mediated reactivation of latent HIV in STAT5 or JNK KO J-Lat 10.6 cells.** Reactivation of HIV-1 eGFP reporter proviruses in NT control (grey) STAT5A, STAT5B, MAPK8 (JNK1) or MAPK9 (JNK2) (green) KO J-Lat 10.6 cells. Cells were pretreated with increasing concentrations of STAT5 or JNK inhibitor or left untreated. Four hs later, the cells were left untreated or treated with TNFα or the indicated concentrations of holo-RBP4. The percentage of cells showing reactivation of HIV-1 eGFP reporter proviruses were determined by flow cytometry. Data are represented as mean ± SEM of two-three independent experiments.


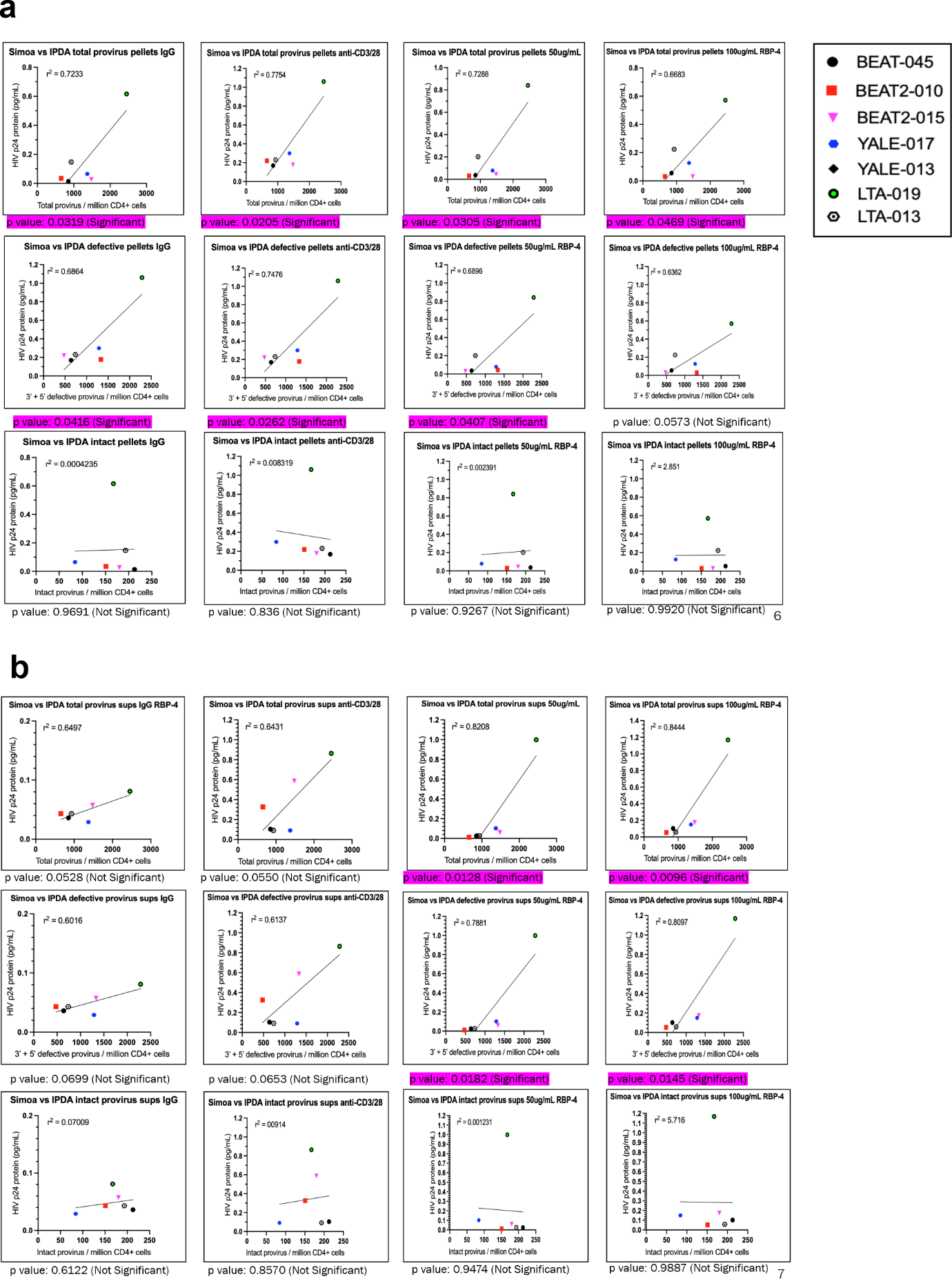


**Figure. S14.**

**Reactivation of latent HIV-1 in PBMCs from PLWH under cART.** (**a**, **b**) Correlation between total (upper panel) or defective (middle panel) or intact (lower panel) provirus/millions of CD4+ T cells from ART-suppressed PLWH and the HIV p24 induction in the cell pellets (**a**) or in the culture supernatants (**b**).

**Table S1.**

Efficiency of Indel Generation calculated with TIDE, indicating CRISPR/Cas9 KO efficiency in J-Lat 10.6 cells.

| Gene | Efficiency of Indel generation |
| --- | --- |
| RELA | 65-81% |
| NFKB1 | 32-55% |
| NIK | 66-72% |
| RELB | 70-72% |
| STRA6 | 56% |
| STAT5A | 52% |
| STAT5B | 82-85% |
| MAPK8 | 87-90% |
| MAPK9 | 81-85% |

**Table S2.**

sgRNAs (5´-3´) used for KO of 10.6 J-Lat cells.

| ACGGAGGCTAAGCGTCGCAA | NT |
| --- | --- |
| TAAGAGGTCAGACCGTCGTG | STATB |
| ACGTGGTGACACGGTACTAC | MAPK9 |
| GGATCAAGCGTGCTGACCGG | STAT5A |
| GAATCAGACTCATGCCAAGC | MAPK8 |
| CTACAAGTACTACTCCCTGC | STRA6_1 |
| CTGGCTGGGACGACATTCTC | STRA6_2 |
| GGGCGGCGTCTTGAACACAA | RELB_1 |
| CAAGTAGAGCTCCTCGCCAC | RELB_2 |
| ACTCGACTACGGCGTCACCG | NFKB2_1 |
| TAGGCTGTTCCACGATCACC | NFKB2_2 |
| GCCTGTTCGCTACGACATGG | NIK_1 |
| TCTGATCAAGACTCTCGGAC | NIK_2 |
| ACATGAGCCGCACCACGCTG | NFKB1_1 |
| GGCACCAGGTAGTCCACCAT | NFKB1_2 |

**Table S3.**

Primers (5´-3´) used for PCR of target genes and verify the KO in 10.6 J-Lat cells.

| TTGCAGTGATTACAGTGGC | STAT5B_Fw |
| --- | --- |
| cacaatctcggttcattacaacc | STAT5B_Rev |
| CTACACTCTCACACTGGC | MAPK9_Fw |
| AACAATATGCAGACCAAC | MAPK9_Rev |
| AGCGAGTGCAGTGGTGAG | STAT5A_Fw |
| AGGCCACCATGCCCAAC | STAT5A_Rev |
| TTGAGCGTCATAGACTTG | MAPK8_Fw |
| CAGTGAATACTGCCATCAC | MAPK8_Rev |
| GCATCTCTGCAGGGCTC | STRA61_Fw |
| CACATCCAACCTAGAGTTG | STRA61_Rev |
| TGCCAGCTTGCTCAGGC | STRA62_Fw |
| ACACTTGGAAACACCACAC | STRA62_Rev |
| CAAAAAAAAAAAATTTTAAAAAGG | RELB1_Fw |
| GTCCTGCCCACTCTG | RELB1_Rev |
| GAGGATCAGATGCCCTGCC | RELB2_Fw |
| ACCTCAGCCTCCCAAATTG | RELB2_Rev |
| GGAGCCAAGCGCCCCC | NFKB21_Fw |
| ACGCCGAGGTCCTGGGCG | NFKB21_Rev |
| GGATGGTATTATTGAATATGATG | NFKB22_Fw |
| CTTGCCCACCAGACTG | NFKB22_Rev |
| ACCTACACTTGAGCCGGG | NIK1_Fw |
| CAGCAGCCTGGAGGGTGAG | NIK1_Rev |
| ATACCAAACCCATTTTATAAGTGAG | NIK2_Fw |
| CAGGGGGCCTCCGTC | NIK2_Rev |
| ACTTTATTAGCAATATGAAG | NFKB11_Fw |
| AGTTTCTCTCTTGATAAAG | NFKB11_Rev |
| TACGGGAAAAGTGATTCTTG | NFKB12_Fw |
| ACATTAAGAGACTGAAGTTTAG | NFKB12_Rev |
